# Intestinal Serotonergic Vagal Signaling as a Mediator of Microbiota-Induced Hypertension

**DOI:** 10.1161/CIRCULATIONAHA.124.072363

**Published:** 2026-09-15

**Authors:** Alan de Araujo, Hemaa Sree Kumar, Tao Yang, Emily B. Otmanowski, Adriana Alviter Plata, Elliott W. Dirr, Nicole R. Bearss, David M. Baekey, Megan R. Dreier, Ivana de la Serna, Darren S. Miller, Basak Donertas-Ayaz, Arashdeep Singh, Andrea L. Kalinoski, Timothy J. Garrett, Brittney Short, Matthew L. Anderson, Christopher J. Martyniuk, Guillaume de Lartigue, Jasenka Zubcevic

**Affiliations:** 1 Monell Chemical Senses Center, University of Pennsylvania, Philadelphia (A.d.A., A.S., G.d.L.).; 2 Department of Neurosurgery, Brain and Spine, University of South Florida, Tampa 7831 (H.S.K., E.B.O., A.A.P., J.Z.).; 3 Microbiomes Institute, University of South Florida, Tampa (H.S.K., E.B.O., A.A.P., J.Z.).; 4 Department of Neurosciences, University of Toledo, OH (H.S.K., N.R.B.).; 5 Department of Physiology and Pharmacology, University of Toledo, OH (T.Y., E.W.D., D.S.M.).; 6 Department of Neuroscience, University of Florida, Gainesville (D.M.B.).; 7 Department of Cell and Cancer Biology, University of Toledo, OH (M.R.D., I.d.l.S.).; 8 Department of Oral and Maxillofacial Surgery, University of Florida, Gainesville (B.D.A.).; 9 Department of Surgery, University of Toledo, OH (A.L.K.).; 10 Department of Pathology, Immunology and Laboratory Medicine, University of Florida, Gainesville (T.J.G.).; 11 Tampa General Hospital Precision Medicine Biorepository, University of South Florida (B.S., M.L.A.).; 12 Department of Physiological Sciences, University of Florida, Gainesville (C.J.M.).

**Keywords:** GI, hypertension, microbiota, serotonin, vagus

## Abstract

**BACKGROUND:**

Hypertension is a pervasive global health challenge, impacting more than 1 billion individuals worldwide. Despite strides in therapeutic strategies, a significant proportion of patients remain resistant to the currently available therapies. Although conventional treatments predominantly focus on cardiac, renal, and cerebral targets, emerging research underscores the pivotal role of the gut and its microbiota. Yet, the precise mechanisms governing interactions between the gut microbiota and the host blood pressure remain unclear.

**METHODS:**

We combined fecal microbiota transplantation between normotensive and hypertensive rats, an intersectional genetic strategy in a serotonin receptor 3a (5HT3a) receptor Cre rat line to activate, ablate, and restore serotonin-sensing colonic vagal neurons, in vivo calcium imaging and radiotelemetric blood pressure recordings, and measurements of intestinal serotonin (5-hydroxytryptamine [5-HT]) and 5HT3a receptor signaling in rats and in colonic biopsies from normotensive and hypertensive human subjects (N=5 per group; 36 to 75 years of age).

**RESULTS:**

A marked decrease in both intestinal 5-HT and vagal 5HT3a receptor signaling was observed in hypertensive rats and humans, and in rats subjected to fecal microbiota transplantation from hypertensive rats. Leveraging the intersectional genetic strategy in a Cre rat line, we demonstrate that intestinal 5HT3a receptor vagal signaling is a crucial link between the gut microbiota and blood pressure homeostasis and that recovery of 5-HT signaling in colon-innervating vagal neurons can alleviate hypertension.

**CONCLUSIONS:**

Here, we describe a neural host-microbiota interaction that is mediated by intestinal serotonin (5-HT) signaling via the vagal 5HT3a, which is crucial for maintenance of blood pressure homeostasis. This finding enhances our comprehension of hypertensive pathophysiology and unveils a promising new therapeutic target for combating resistant hypertension associated with gut dysbiosis.

**Clinical Perspective**

**What Is New?**

- Colon-innervating vagal sensory neurons that detect intestinal serotonin through serotonin receptor 3a receptors are necessary and sufficient for blood pressure homeostasis.
- Hypertensive gut microbiota epigenetically suppresses colonic tryptophan hydroxylase 1 and serotonin production, blunting this serotonergic vagal signal and raising blood pressure, and this is reversible by microbial replacement.
- Humans with diagnosed hypertension show reduced colonic serotonin, tryptophan hydroxylase 1, and serotonin receptor 3a receptor expression, as in hypertensive rats.

**What Are the Clinical Implications?**

- The intestinal serotonergic vagal axis is a peripherally accessible, reversible regulator of blood pressure.
- This microbiota-gut–brain pathway offers a novel therapeutic target for resistant hypertension not addressed by current cardiac-, renal-, or central-acting drugs.
- Microbiota-, dietary-, and neuromodulation-based strategies that restore this pathway warrant study as adjuncts for resistant hypertension.

Blood pressure is of fundamental importance for the fitness of the whole organism, and chronically elevated blood pressure or hypertension is a leading cause of cardiovascular, renal, and neurological conditions when left untreated.^1–3^ Despite its growing global prevalence, now affecting >1.5 billion individuals, hypertension remains therapeutically challenging because of its heterogeneous etiology and the high incidence of treatment resistance.^4–6^ These limitations underscore the need to identify novel physiological mechanisms that regulate blood pressure and may be therapeutically targeted.

Recent work has highlighted gut microbiota as an important contributor to blood pressure regulation. The gastrointestinal (GI) tract harbors a dense and diverse ecosystem of microbes that influence various physiological processes including cardiovascular function. Several studies have reported associations between gut dysbiosis, a term that describes an imbalance in gut microbes, and high blood pressure in rodents and human patients.^7–9^ However, whether microbial signals are actively sensed by the host neural circuits, and the identity of the sensory pathways through which they influence blood pressure, remains poorly defined.

Building on our previous findings that electric stimulation of the subdiaphragmatic (GI) vagus nerve ameliorated development of hypertension in rodent models of gut dysbiosis,^10^ we hypothesized that impaired GI vagal sensory signaling contributes to microbiota-induced hypertension. Emerging evidence suggests that intestinal serotonin (5-hydroxytryptamine [5-HT]) plays a key role in gut–brain signaling,^11–13^ raising the possibility that serotonergic signaling represents a mechanistic link between the gut microbiota and cardiovascular control.

Here, we investigated whether intestinal serotonergic vagal signaling constitutes a causal pathway through which the gut microbiota regulates blood pressure. Specifically, we sought to identify the host sensory neuron populations that detect microbiota-derived intestinal cues, determine how dysbiosis alters their signaling, and test whether restoring this communication is sufficient to alleviate hypertension. We further aimed to evaluate the translational relevance of this pathway by investigating intestinal 5-HT signaling in humans with diagnosed hypertension. Together, these experiments address whether a gut-derived serotonergic vagal pathway represents a previously unrecognized and therapeutically actionable mechanism linking the microbiota to blood pressure dysregulation.

## METHODS

Detailed experimental procedures are provided in the Expanded Methods.

### Institutional Approval

All human procedures were approved by the University of South Florida institutional review board (STUDY000356) and conducted in accordance with the Declaration of Helsinki; written informed consent was obtained from all participants. All animal procedures were approved by the institutional animal care and use committees of the University of Toledo, the University of South Florida, and the University of Florida.

### Data Sharing and Availability

16S sequencing data are available at NCBI BioProject PRJNA1140871 (http://www.ncbi.nlm.nih.gov/bioproject/1140871). Untargeted and targeted metabolomics data are deposited at the National Institutes of Health Common Fund Metabolomics Workbench (Metabolomics Data Repository and Coordinating Center; www.metabolomicsworkbench.org) under REQ20260618220704. Normalized metabolite measures are provided in Table S7. Additional data and code are available upon request to the corresponding authors.

### Statistical Analysis

Sample sizes were determined a priori by power analysis (80% power; α=0.05) based on the expected blood pressure effect size (Cohen’s *d*) from our previous work.^10,14^ Animals were randomly assigned to treatment groups. Whenever feasible, data collection and analyses were performed by a blinded experimenter. The experimental unit was the individual animal throughout; when multiple measurements were obtained per animal (eg, left and right nodose ganglia [NG]), values were averaged so that the animal, not the ganglion or neuron, served as the unit of analysis. A total of 120 animals were used in this study; per-experiment group sizes are reported in each Figure legend. After testing for normality (Shapiro-Wilk test), intergroup differences were analyzed by unpaired or paired, 2-tailed Student *t* test for single comparison, or by one-way or 2-way ANOVA with an appropriate multiple-comparison test, as indicated in the Figure legends. If nonparametric testing was indicated, intergroup differences were analyzed by Mann-Whitney *U* test or Kruskal-Wallis test with Dunn’s correction, as appropriate. The *F* test for equality of variances was performed and, if indicated, appropriate corrections were made (Welch’s correction for the *t* test) using Prism 9 (GraphPad). Longitudinal telemetry data (repeated blood pressure measurements in the same animals over time; Figure 1C; Figure 3I; Figure 5F; Figure 6D) were analyzed using a mixed-effects model (restricted maximum likelihood) equivalent to a 2-way repeated-measures ANOVA, with treatment (group) and time as factors and a random intercept for individual animal to account for within-subject correlation; significant main effects or interactions were followed by Sidak-corrected multiple comparisons at each time point. The statistical reporting in this work follows the ARRIVE (Animal Research: Reporting of In Vivo Experiments) 2.0 guidelines.^15^ Data are presented as mean±SEM.

**Figure 1.**
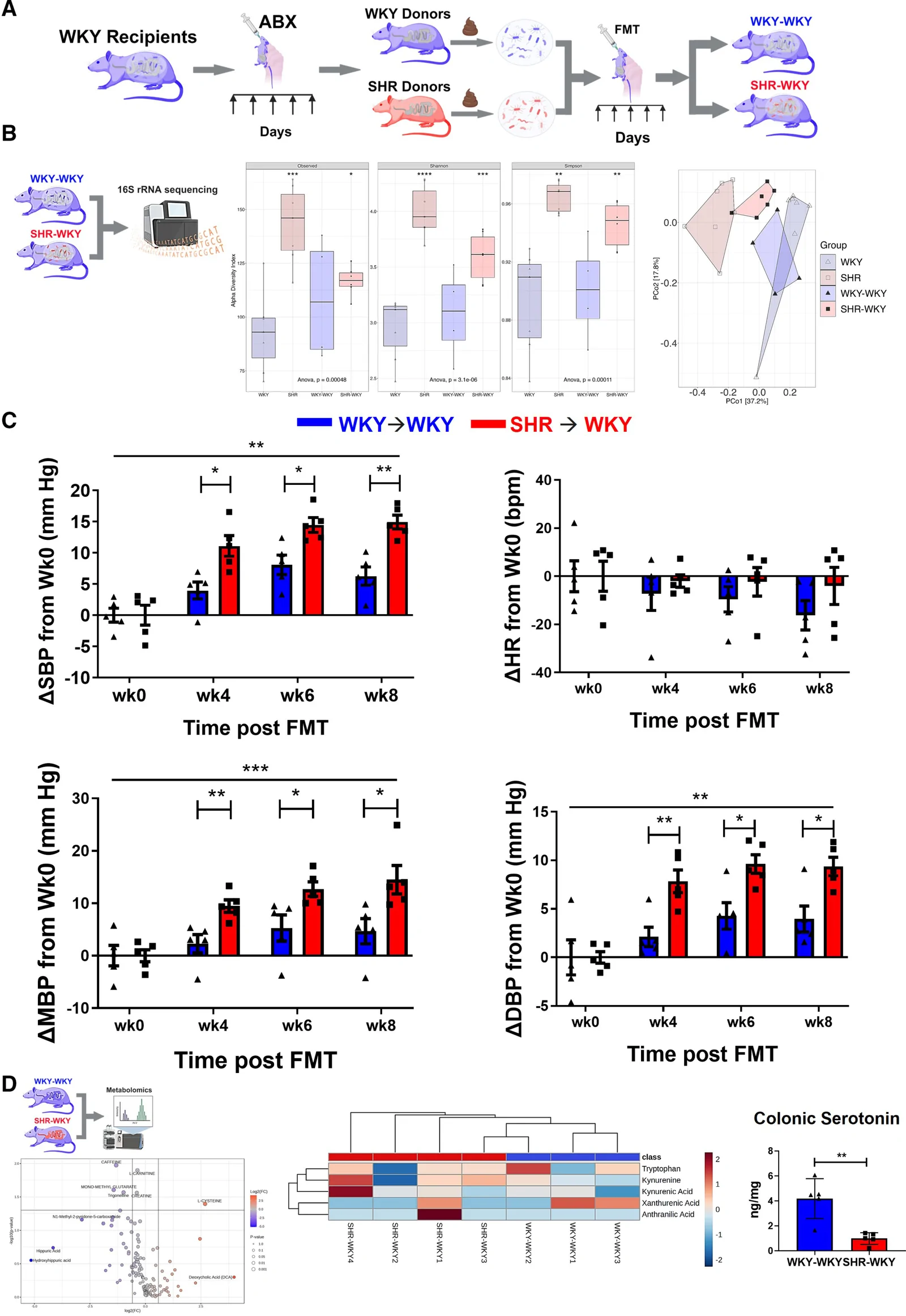
**Transplant of hypertensive microbiota increased blood pressure and reduced colonic serotonin. A**, Cartoon schematics illustrating fecal microbiota transplant (FMT). Normotensive Wistar Kyoto (WKY) rats were administered a cocktail of antibiotics (ABX) by daily oral gavage for 5 days, followed by oral gavage of fecal slurry from either the normotensive WKY or the spontaneously hypertensive rat (SHR) donors for 5 consecutive days, then once per week for 8 weeks (wk; 1:1 donor to recipient ratio). Two groups were generated: WKY→WKY (blue) and SHR→WKY (red), reflecting donor→recipient microbiota transplant. **B**, **Left**, Schematics of end point 16S bacterial sequencing performed in all donor and recipient groups (N=4–7 per group). **Middle** and **far right**, Alpha and beta diversity plots after 16S bacterial sequencing analyses show bacterial abundance and diversity highlighting statistical similarities between the WKY and SHR donors and their respective gut bacteria recipient groups. Values are mean±SEM; one-way ANOVA followed by Sidak’s multiple-comparisons test (or Kruskal-Wallis with Dunn’s correction in which data did not meet normality); *P<0.05; **P<0.01; ***P<0.001. **C**, Radiotelemetric measurements of continuous systolic, diastolic, and mean blood pressure (SBP, DBP, and MBP; mm Hg) and heart rate (HR; beats per minute [bpm]) were performed once per week over 24 hours in conscious unrestrained WKY→WKY (blue) and SHR→WKY (red) rats at baseline (ie, before FMT, noted as wk0) and then at weeks 4 through 8. To better illustrate change from baseline (wk0) for each rat and group, weekly absolute values were normalized to values observed at baseline (wk0) for each rat and presented as change (Δ) from wk0. **Top left**, A significant difference in post-FMT increase in SBP from wk0 was observed in the SHR→WKY group (red) compared with the WKY→WKY control group (blue) starting 4 weeks after FMT (N=5 per group). **Top right**, No major differences were observed in HR between the groups. **Bottom left**, A significant difference in post-FMT MBP increase was observed between WKY→WKY and SHR→WKY starting 4 weeks after FMT. **Bottom right**, A significant difference in post-FMT increase in DBP was observed between WKY→WKY and SHR→WKY starting at 4 weeks after FMT. Line across all time points reflects a significant interaction between treatment and time. Values are mean±SEM; mixed-effects model (restricted maximum likelihood [REML]), with treatment and time as factors and animal as a random effect, followed by Sidak’s multiple-comparisons test; *P<0.05; **P<0.01. **D**, **Left,** Cartoon schematics of end point metabolomics performed in proximal colons of WKY→WKY (blue) and SHR→WKY (red) rats using liquid chromatography coupled with mass spectrometry (LC-MS/MS). The volcano plot below reflects significant divergence of overall metabolites between the WKY→WKY (blue) and SHR→WKY (red) reflected in the volcano plot and heat map (N=3–4 per group) as measured by untargeted metabolomics. **Middle**, Targeted metabolomics for tryptophan (Trp) metabolites shows no difference in detected Trp metabolites between the WKY→WKY (blue) and SHR→WKY (red; N=3–4 per group). **Far right**, An ≈4-fold decrease in serotonin (5-HT) in the proximal colons of SHR→WKY (blue) compared with WKY→WKY (red; N=5 per group) as measured by ELISA. Values are mean±SEM; Student *t* test; **P<0.01. Created in BioRender. Sree, H. (2026) https://BioRender.com/yiop4ui.

**Figure 2.**
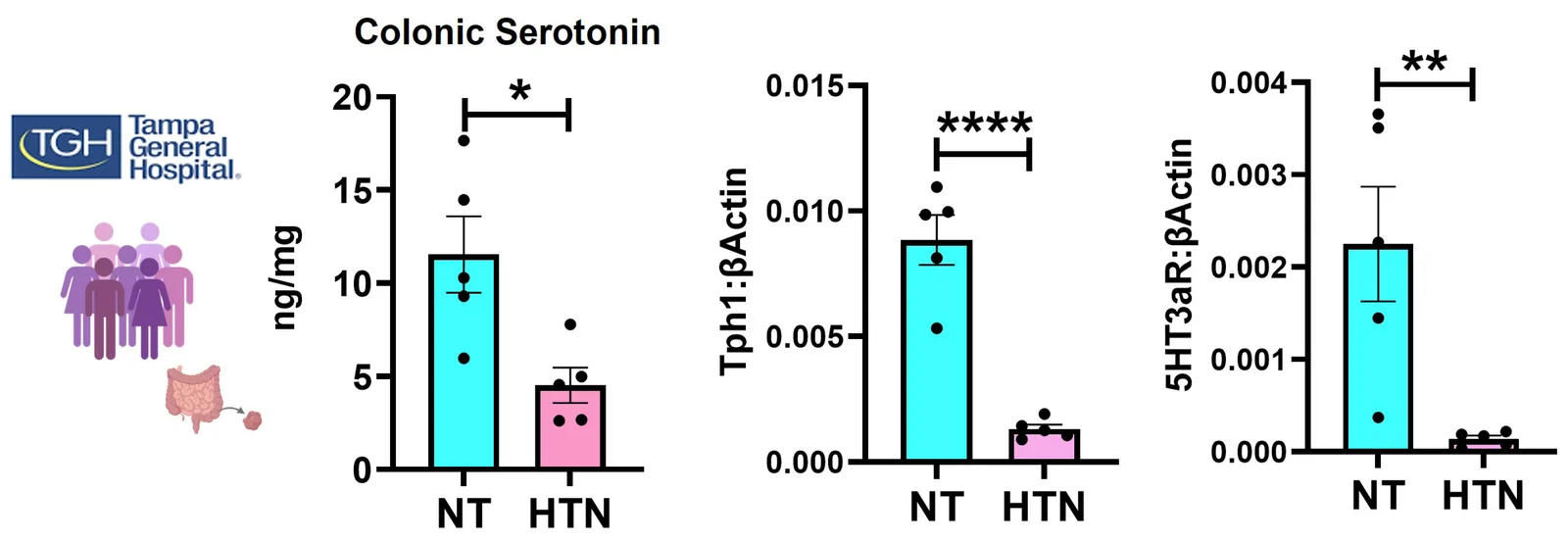
**Reduced serotonin in colonic biopsy samples from human subjects with diagnosed hypertension. Left**, Serotonin levels were measured by commercial ELISA assay in colonic biopsy samples obtained from human normotensive (NT) and hypertensive (HTN) subjects (N=5 per group). Samples were obtained with written informed consent via Tampa General Hospital Biorepository (institutional review board STUDY000356). **Middle and far right**, Reduced expression levels of tryptophan hydroxylase 1 (Tph1) and serotonin receptor 3a (5HT3a) receptors (5HT3aR) in the colons of HTN human subjects. Values are mean±SEM; Student *t* test, *P<0.05; **P<0.01; ****P<0.0001.

**Figure 3.**
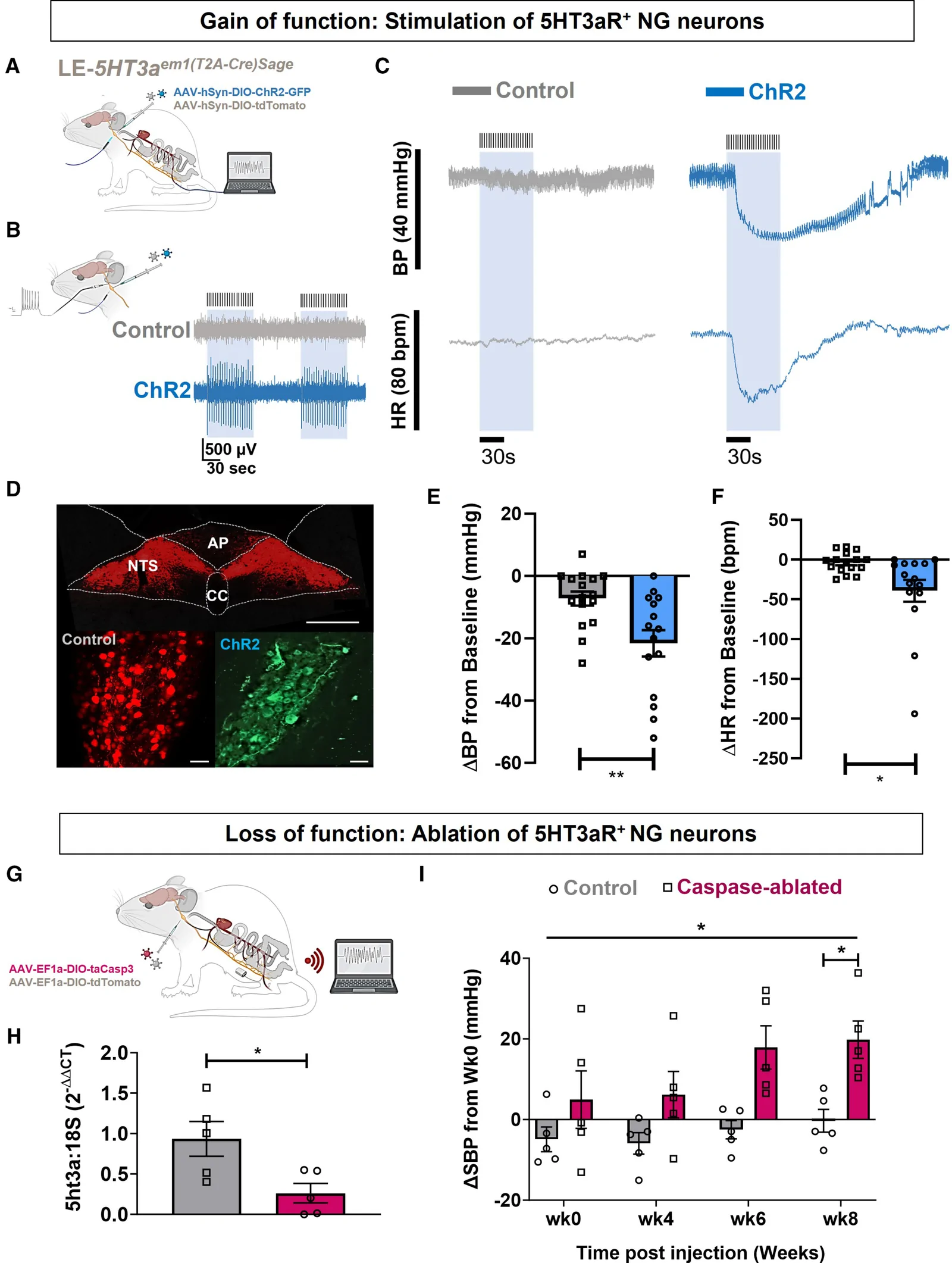
**Vagal afferents sensing serotonin can modify blood pressure. A**, Cre-dependent viral vectors (adeno-associated virus [AAV]) expressing either excitatory channelrhodopsin 2 (ChR2) or control tdTomato (Control) were bilaterally microinjected in the nodose ganglia (NG) of LE-5HT3a^em1(T2A-Cre)Sage^ (5HT3aR^Cre^) rats. Three weeks (wk) later, rats were anesthetized and femoral artery was cannulated for measurement of direct blood pressure (BP; mm Hg) and heart rate (HR; beats per minute [bpm]) before, during, and after application of the blue excitatory light to NG for 1 minute. **B**, An example of optic stimulation of the NG expressing the excitatory ChR2 (in blue) and the Control tdTomato (Control; in gray). Blue light stimulation produced a time-locked activation of vagal afferents expressing only the excitatory ChR2 in 5HT3aR^Cre^ rats. Light blue box illustrates timing of optic stimulation. **C**, Representative traces of BP and HR (beats per minute [bpm]) before, during, and after optic stimulation of the NG expressing the excitatory ChR2 or Control tdTomato. Optic stimulation of NG expressing ChR2 produced a time-locked decrease in BP and HR (in blue), whereas optic stimulation of the NG expressing the Control tdTomato (in gray) did not produce a significant response in 5HT3aR^Cre^ rats. Light blue box illustrates timing of optic stimulation. **D**, End point confirmation of viral expression of Control (tdTomato; in red) in the nucleus of the solitary tract (NTS; **top**; scale bar=500 µm) and NG (**bottom left**), and ChR2 (in green) in the NG (**bottom right**; scale bar=100 µm). AP indicates area postrema; and CC, central canal as per Paxinos and Watson Rat Brain Atlas. **E**, Average BP (mm Hg) responses to bilateral optic stimulation of the NG expressing ChR2 (blue bar) and Control (tdTomato, gray bar; N=8 rats per group). Each rat contributed 2 measurements (left and right NG stimulated and recorded separately), yielding 16 data points; statistics were computed with the rat as the experimental unit by averaging the left and right responses. Absolute BP values are normalized to values observed at baseline for each rat and presented as change (Δ) from baseline. Values are mean±SEM; Student *t* test; **P<0.01. **F**, Average HR (bpm) responses to bilateral optic stimulation of the NG expressing ChR2 (blue bar) and Control (tdTomato; gray bar; N=8 rats per group). Absolute HR values are normalized to values observed at baseline for each rat and presented as change (Δ) from baseline. Values are mean±SEM; Student *t* test; *P<0.05. **G**, Radiotelemetry transmitters were implanted in femoral artery of 5HT3aR^Cre^ rats for continuous measurement of BP and HR in conscious unrestrained rats. After baseline measurements of BP and HR (wk0), Cre-dependent viral vectors (AAV) expressing either Caspase 3 (Cas; magenta), to selectively ablate 5HT3a receptor (5HT3aR)–expressing vagal afferents, or Control tdTomato (gray) were bilaterally microinjected in the NG in separate 5HT3aR^Cre^ rats. **H**, At end point, virally mediated ablation of 5HT3a receptor–expressing vagal afferent neurons was confirmed by reduced relative expression levels of NG 5HT3a receptors in Cas-injected rats (magenta), as measured by quantitative real-time PCR (RT-qPCR; N=5 per group). Values are mean±SEM; Student *t* test; *P<0.05. **I**, Radiotelemetric measurements of continuous systolic BP (SBP; mm Hg) were performed once per week over 24 hours in conscious unrestrained 5HT3aR^Cre^ rats at baseline (ie, before NG viral injections, noted as wk0) and then at weeks 4 through 8. A significant increase in SBP (by ≈18 mm Hg) was observed in the Cas-injected group (magenta) compared with the Control group (gray) 8 weeks after injection (N=5 per group). Line across all time points reflects significant interaction between treatment and time. Absolute values are normalized to values observed at baseline for each rat and presented as change (Δ) from baseline. Values are mean±SEM; mixed-effects model (restricted maximum likelihood [REML]), with treatment and time as factors and animal as a random effect, followed by Sidak’s multiple-comparisons test; *P<0.05. LE indicates Long-Evans. Created in BioRender. Sree, H. (2026) https://BioRender.com/69fsbo0.

**Figure 4.**
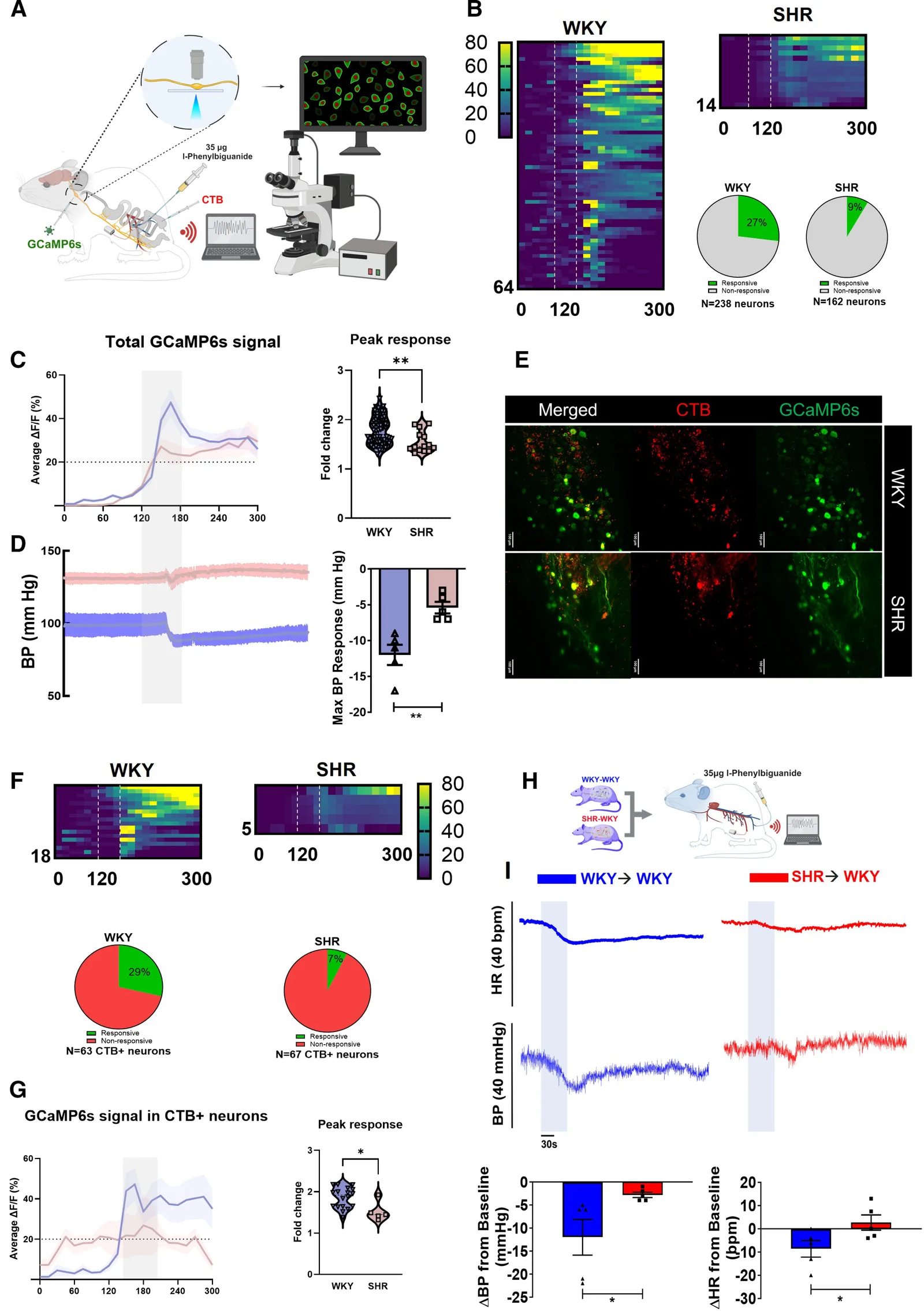
**Reduced serotonin signaling by intestinal vagal afferents in hypertensive rodents with gut dysbiosis. A**, Cartoon schematic illustrating in vivo recordings of calcium responses in vagal afferents co-expressing GCaMP6s (green fluorescent protein-calmodulin-M13 peptide sequence) and the red fluorescent cholera toxin B (CTB) expressed retrogradely from the proximal colon, in response to IV infusion of 5HT3a receptor agonist L-phenylbiguanide (35 µg). Blood pressure (BP) responses were measured simultaneously with neuronal responses in all rats. **B**, The heat maps depict *z*-score–normalized fluorescence traces from all GCaMP6s^+^ vagal neurons in the nodose ganglia (NG) of normotensive Wistar Kyoto (WKY) rats (**left**) and spontaneously hypertensive rats (SHRs; **right**), responding to IV injection of the serotonin receptor agonist. Each row represents the activity of a single cell over 5 minutes. Stimulus was given at 120 seconds (white box). In the N=5 per group, 27% of the total of 238 GCaMP6s-expressing neurons responded to serotonin agonist in the WKY rats, with only 9% of the total of 162 GCaMP6s-expressing neurons responsive in the SHRs, as depicted by pie charts. The responses of each neuron are represented in the heat maps. **C**, **Left**, Total GCaMP6s^+^ neuronal responses are normalized to values observed at baseline (F_0_) for each rat and presented as change (Δ) from baseline (%increase in activity). Stimulus was given at 120 seconds (white box). **Right**, Average peak amplitudes of vagal responses were reduced in the SHRs (N=5 per group). Values are mean±SEM; Student *t* test; **P<0.01. Individual responsive neurons or per-ganglion responses are plotted; statistics were computed with the rat as the experimental unit, with values averaged across left and right stimulation per animal to avoid pseudo-replication. **D**, **Left**, BP responses were time-locked to the calcium responses of total GCaMP6s-expressing vagal neurons (in **C** above). Stimulus was given at 120 seconds (white box). **Right**, Average values of maximum BP (mm Hg) responses to infusion of serotonin receptor agonist in anesthetized WKY (blue) and SHRs (red; N=6 per group). A significantly diminished BP response was observed in the SHRs compared with the WKY rats. Absolute values are normalized to values observed at baseline for each rat and presented as change (Δ) from baseline. Values are mean±SEM; Student *t* test; **P<0.01. **E**, Image depicting colon-projecting vagal afferents retrogradely labeled by CTB (in red) and total GCaMP6s-expressing NG neurons (in green) showing a similar population of neurons in WKY and SHR (also quantified in the pie charts in **F**). Scale bar=100 µm. **F**, Heat maps depict *z*-score–normalized fluorescence traces only from colon-projecting GCaMP6s^+^ vagal neurons in the NG of normotensive WKY rats (**left**) and SHRs (**right**), responding to IV infusion of serotonin receptor agonist. Each row represents the activity of a single cell over 5 minutes. Stimulus was given at 120 seconds (white box). In the N=5 per group, a similar population of neurons is double labeled in the WKY rats and SHRs, and 29% of the total of the 63 colon-projecting GCaMP6s-expressing neurons responded to the serotonin receptor agonist in the WKY rats, with only 7% of the 67 colon-projecting GCaMP6s-expressing neurons responsive in the SHRs, as depicted by pie charts. The responses of each neuron are represented by heat maps. **G**, **Left**, Average traces of calcium responses of GCaMP6s-expressing vagal neurons projecting to the colon in anesthetized WKY (blue) and SHR (red; N=6 per group) in response to IV infusion of serotonin receptor agonist L-phenylbiguanide. Absolute values are normalized to values observed at baseline (F_0_) for each rat and presented as change (Δ) from baseline (%increase in activity). **Right**, Average peak amplitudes of colon-projecting vagal afferents were reduced in the SHRs (red; N=5 per group). Values are mean±SEM; Student *t* test; *P<0.05. Individual responsive neurons or per-ganglion responses are plotted; statistics were computed with the rat as the experimental unit, with values averaged across left and right stimulation per animal to avoid pseudo-replication. **H**, Cartoon schematic illustrating BP (mm Hg) and HR (beats per minute [bpm]) measurements in anesthetized WKY→WKY (blue) and SHR→WKY rats (red) in response to IV infusion of serotonin receptor agonist L-phenylbiguanide. **I**, **Top**, Representative traces of BP and HR responses in both groups, with shaded bar representing length of infusion. **Bottom**, Average BP (**left**) and HR (**right**) peak responses, significantly reduced in the SHR→WKY group (N=5 per group). Absolute values are normalized to values observed at baseline for each rat and presented as change (Δ) from baseline. Values are mean±SEM; Student *t* test; *P<0.05. Created in BioRender. Sree, H. (2026) https://BioRender.com/69fsbo0.

**Figure 5.**
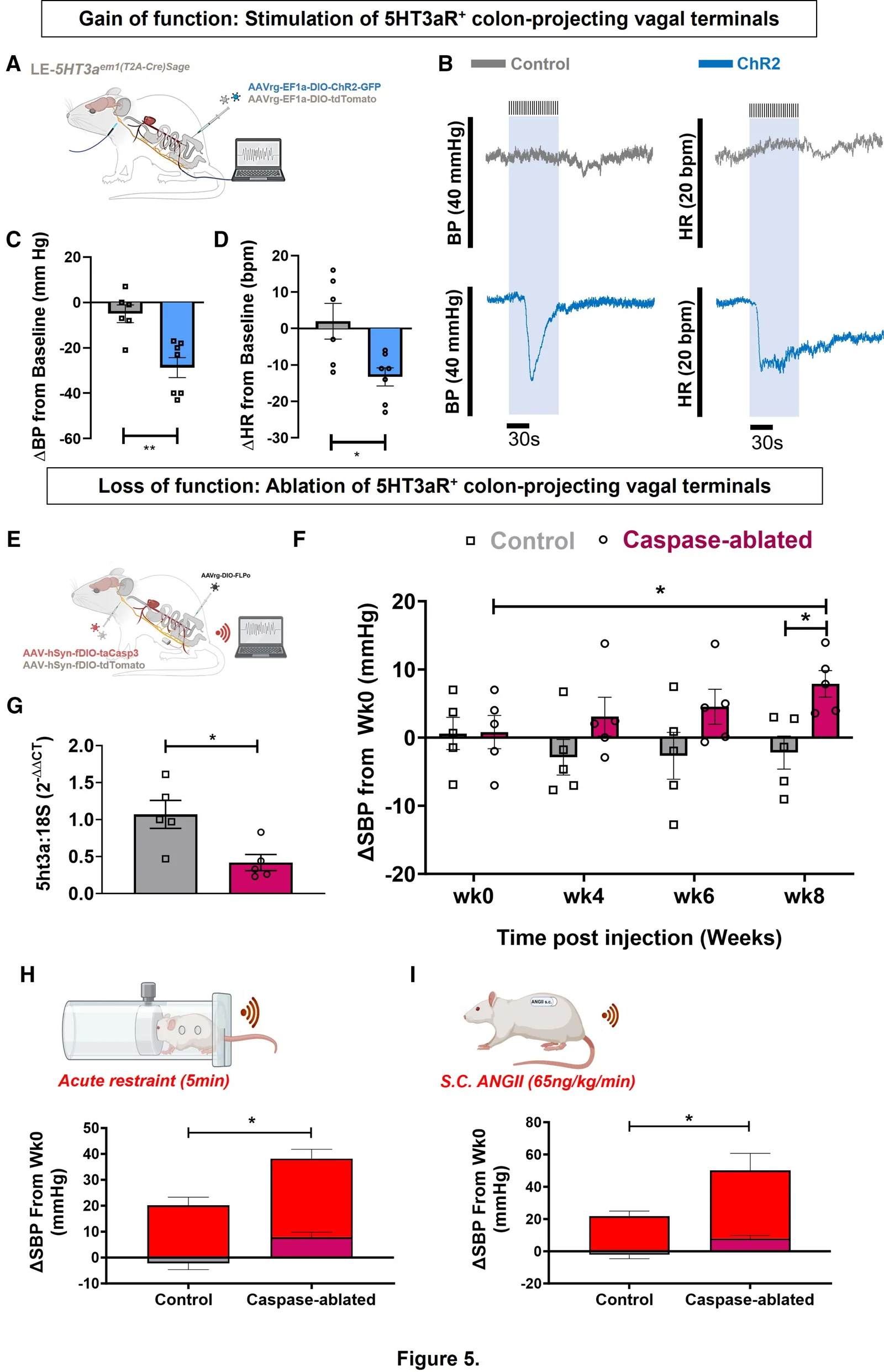
**Colonic vagal serotonin signaling mediates blood pressure homeostasis and stress response. A,** Retrograde Cre-dependent viral vectors (retrograde adeno-associated virus [AAVrg]) expressing either excitatory channelrhodopsin 2 (ChR2) or Control tdTomato fluorescent protein were microinjected in the proximal colons of LE-5HT3a^em1(T2A-Cre)Sage^ (5HT3aR^Cre^) rats. Three weeks later, rats were anesthetized, and femoral artery was cannulated for measurement of direct blood pressure (BP; mm Hg) and heart rate (HR; beats per minute [bpm]) before, during, and after application of the blue excitatory light to the nodose ganglia (NG) for 1 minute. **B**, Representative traces of BP and HR before, during, and after the optic stimulation of the NG. Optic stimulation of the NG expressing ChR2 produced a time-locked decrease in BP and HR (in blue), whereas optic stimulation of the Control NG (in gray) did not produce any significant responses in 5HT3aR^Cre^ rats. Light blue box illustrates timing of optic stimulation. **C**, Average BP (mm Hg) responses to bilateral optic stimulation of the NG expressing ChR2 (blue bar) and Control (gray bar; N=6–7 rats per group). Absolute BP values after stimulation of each NG separately are normalized to values observed at baseline, averaged for each rat, and presented as change (Δ) from baseline. Values are mean±SEM; Student *t* test; **P<0.01. **D**, Average HR (bpm) responses to bilateral optic stimulation of the NG expressing ChR2 (blue bar) and Control (gray bar; N=6–7 rats per group). Absolute HR values after stimulation of each NG separately are normalized to values observed at baseline, averaged for each rat, and presented as change (Δ) from baseline. Values are mean±SEM; Student *t* test, *P<0.05. **E**, Radiotelemetry transmitters were implanted in femoral artery of 5HT3aR^Cre^ rats for continuous measurement of BP and HR in conscious unrestrained rats. At the same time, a retrograde Cre-dependent vector (AAVrg) expressing FLPo (Flp recombinase, optimized) recombinase was injected in colons of all rats. After recovery and baseline BP recordings (wk0), FLPo-dependent vectors expressing either Caspase 3, to selectively ablate 5HT3a receptor-expressing vagal afferents that project to the colon only, or Control YFP (yellow fluorescent protein) protein were bilaterally microinjected in the NG of separate 5HT3aR^Cre^ rats. **F**, Radiotelemetric measurements of continuous systolic BP (SBP; mm Hg) were performed once per week over 24 hours in conscious unrestrained 5HT3aR^Cre^ rats at baseline (ie, before NG viral injections, noted as wk0) and then at weeks 4 through 8. A significant increase in SBP (by ≈9 mm Hg) was observed in the Caspase-ablated rats (magenta) compared with Control group (gray) and compared with its baseline (wk0) 8 weeks after injection (N=5 per group). Absolute values are normalized to values observed at baseline for each rat and presented as change (Δ) from baseline. Values are mean±SEM; mixed-effects model (restricted maximum likelihood [REML]) with treatment and time as factors and animal as a random effect, followed by Sidak’s multiple-comparisons test; *P<0.05. **G**, At end point, virally mediated ablation of 5HT3a receptor–expressing colonic vagal neurons was confirmed by reduced relative expression levels of NG 5HT3a receptors in Caspase-ablated rats (magenta) as measured by quantitative real-time PCR (RT-qPCR; N=5 per group). Values are mean±SEM; Student *t* test; *P<0.05. **H**, BP responses to 5 minutes of acute restraint were measured by radiotelemetry in both sets of rats 8 weeks after injection. 5HT3a^Cre^ rats with Caspase-ablated 5HT3a receptor-expressing colonic vagal neurons (magenta) presented with significantly higher SBP (mm Hg) responses to the acute restraint compared with the Control group (gray). Absolute values are normalized to values observed at w0 for each rat and presented as change (Δ) from wk0, with red bars representing additive effects of restraint at week 8 (wk8). Values are mean±SEM; mixed-effects model (REML) with treatment and time as factors and animal as a random effect, followed by Sidak’s multiple-comparisons test; *P<0.05. **I**, BP responses to a 1-week subcutaneous angiotensin II (s.c. ANGII) infusion were measured by radiotelemetry in both groups starting from 8 weeks after injection. 5HT3a^Cre^ rats with Caspase-ablated 5HT3a receptor-expressing colonic vagal neurons (magenta) presented with significantly higher SBP (mm Hg) responses to 1 week of s.c. ANGII infusion compared with the Control group (gray). Absolute values are normalized to values observed at 8 weeks for each rat and presented as change (Δ) from wk8, with red bars representing additive effects of ANGII. Values are mean±SEM; mixed-effects model (REML) with treatment and time as factors and animal as a random effect, followed by Sidak’s multiple-comparisons test; *P<0.05. LE indicates Long-Evans. Created in BioRender. Sree, H. (2026) https://BioRender.com/69fsbo0.

**Figure 6.**
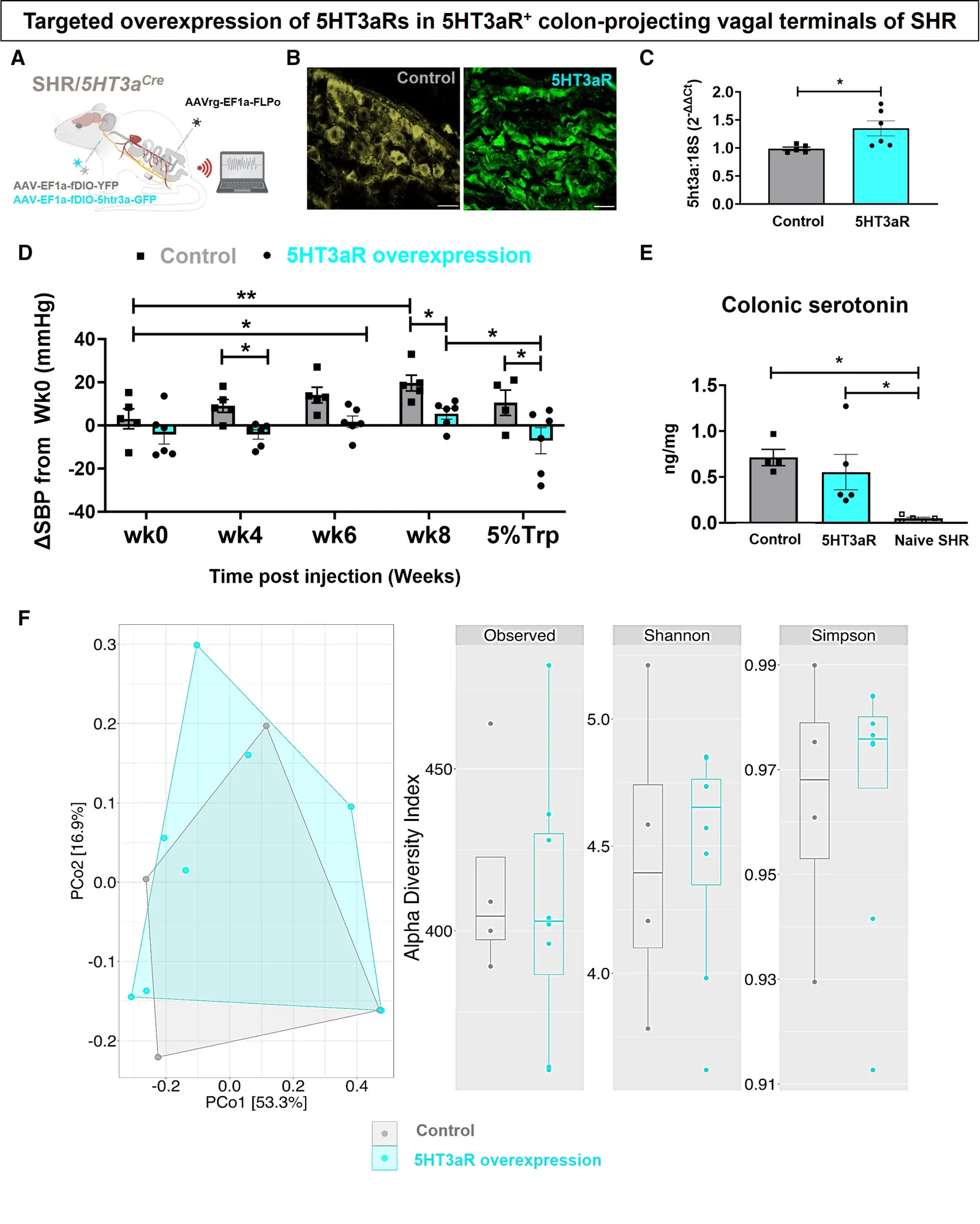
**Restoring gut vagal serotonin signaling alleviates microbiota-induced hypertension. A**, Radiotelemetry transmitters were implanted in femoral artery of hypertensive male and female SHR/5HT3aR^Cre^ rats for continuous measurement of blood pressure (BP; mm Hg) and heart rate (HR; beats per minute [bpm]) in conscious unrestrained rats. At the same time, a retrograde Cre-dependent vector (retrograde adeno-associated virus [AAVrg]) expressing FLPo (Flp recombinase, optimized) recombinase was injected in colons of all rats. After recovery and baseline BP recordings (wk0), FLPo-dependent vectors (AAV) expressing either the 5HT3a receptors or control YFP fluorescent protein were bilaterally microinjected in the nodose ganglia (NG) in separate rats. **B**, End point confirmation of viral expression of control YFP (in yellow) and 5HT3a receptor (in green) in the NG of SHR/5HT3aR^Cre^ rats. Scale bar=100 µm. **C**, At end point, virally mediated overexpression of 5HT3a receptors was confirmed by quantitative real-time PCR (RT-qPCR) in the NG (N=6 per group). Values are mean±SEM; Student *t* test; *P<0.05. **D**, Radiotelemetric measurements of continuous systolic BP (SBP; mm Hg) were performed once per week over 24 hours in conscious unrestrained SHR/5HT3aR^Cre^ rats at baseline (ie, before NG viral injections, noted as wk0) and then at weeks 4 through 8. Expression of 5HT3a receptors (cyan) in colonic vagal neurons significantly alleviated time-dependent increase in SBP compared with the Control group (gray). This effect was potentiated by dietary supplementation with 5% tryptophan (Trp) administered for 1 week ad libitum, but only in rats with 5HT3a receptor overexpression (in cyan). Absolute values are normalized to values observed at baseline for each rat and presented as change (Δ) from baseline (N=4–6 per group). Values are mean±SEM; mixed-effects model (restricted maximum likelihood [REML]) with treatment and time as factors and animal as a random effect, followed by Sidak’s multiple-comparisons test, *P<0.05, **P<0.01. **E**, One week–long supplementation with 5% Trp in diet elevated colonic serotonin in both the Control (in gray) and 5HT3a receptor overexpression (in cyan) groups compared with the naïve SHR (light red; open squares; N=5 per group). Values are mean±SEM; one-way ANOVA followed by Sidak’s multiple-comparisons test (or Kruskal-Wallis with Dunn’s correction in which data did not meet normality; *P<0.05). **F**, End point 16S bacterial sequencing analyses show an overlap in composition and abundances of the gut bacteria between the 2 groups, illustrated by the principal coordinate analysis (PCoA) showing beta diversity with Bray-Curtis Dissimilarity (**left**) and Alpha diversity plots with the common indices (**right**; N=4–6 per group). Created in BioRender. Sree, H. (2026) https://BioRender.com/69fsbo0.

## RESULTS

### Fecal Transplant of Hypertensive Microbiota Reduces Colonic Serotonin and Elevates Blood Pressure

Previous studies have associated gut dysbiosis with hypertension,^7–9,16,17^ yet the precise mechanisms by which microbial signals influence blood pressure remain unclear. To determine the mechanisms by which hypertensive microbiota cause hypertension, we employed fecal microbiota transplantation (FMT) in normotensive Wistar Kyoto (WKY) rats. Endogenous gut bacteria were first depleted by administering a 5-day course of oral antibiotics,^18^ resulting in a significant decrease in fecal biomass (Figure S1A). Antibiotic-treated WKY rats then received whole microbiota transplants from donor rats, which were either spontaneously hypertensive rats (SHRs) or their normotensive genetic controls, the WKY rats (Figure 1A). Successful engraftment of donor microbiota was confirmed by 16S rRNA gene sequencing at the experimental end point of 8 weeks (Figure 1B). We found that the microbial community composition in FMT recipients closely resembled that of their respective donors, as illustrated by the box and principal component plots highlighting alpha and beta diversity (Figure 1B), indicating stable colonization after transplantation.

Continuous telemetric recordings revealed a progressive, time-dependent rise in blood pressure in the SHR→WKY group compared with the WKY→WKY controls (Figure 1C and Figure S1F). Blood pressure elevation became evident within weeks after transplantation with significant differences observed 8 weeks after FMT, whereas heart rate remained unchanged between groups within this time (Figure 1C; Figure S1F). These findings are consistent with previous reports linking gut dysbiosis to hypertension in rodent models^17,19^ and establish that hypertensive microbiota is sufficient to drive sustained increases in blood pressure in normotensive hosts.

The proximal colon is the primary site of host-microbiota interactions.^20^ To determine whether hypertensive microbiota alter colonic metabolic pathways, we first performed untargeted metabolomic profiling of proximal colon tissue from WKY→WKY and SHR→WKY FMT recipients, at the time point when blood pressure differences were maximal (8 weeks after FMT), using high-resolution liquid chromatography coupled with mass spectrometry (Figure 1D; Table S4). This nontargeted analysis revealed broad alterations in the colonic metabolite landscape after transplantation of hypertensive microbiota (Figure 1D, volcano plot). Previous studies reported disrupted intestinal tryptophan (Trp) metabolism in hypertension in both humans and rodents,^21–23^ and we tested this in FMT and donor colons using targeted metabolomics. We detected several Trp metabolites using our approach (Figure 1D, middle; Tables S4 and S5). We observed a similarity in the levels of Trp metabolites detected by liquid chromatography coupled with mass spectrometry in colons of FMT groups (Figure 1D; Table S4) and that of their donors (Table S5). Although 5-HT is a major Trp-derived signaling molecule in the GI tract,^24,25^ it is not reliably detected by standard metabolomics approaches because of its chemical properties and low ionization efficiency^26^ (Table S5). We therefore quantified colonic 5-HT using a validated and optimized ELISA assay, while relying on liquid chromatography coupled with mass spectrometry to assess global Trp metabolites. Targeted 5-HT ELISA measurements revealed an approximately 4-fold reduction in colonic 5-HT levels in SHR→WKY recipients compared with WKY→WKY controls (Figure 1D, far right). This reduction mirrored differences observed in colonic tissue from naïve SHR and WKY donors (Figure S1C). Importantly, fecal Trp and 5-HT levels did not differ between WKY→WKY and SHR→WKY recipients (Figure S1E, far right; Table S6), despite a difference in overall fecal metabolites (Figure S1E, left and middle; Table S6), indicating that the observed reduced colonic 5-HT reflected impaired serotonin synthesis and signaling pathways in the host that is not attributable to altered luminal Trp availability or microbial-derived 5-HT.

To determine whether this microbiota-driven repression of colonic 5-HT signaling is reversible and functionally linked to blood pressure regulation, we performed reverse FMT by transplanting microbiota from normotensive WKY donors into SHR recipients (WKY→SHR), with SHR→SHR group serving as transplant controls (Figure S2A). Transplantation of normotensive microbiota into hypertensive SHR recipients was successful and it restored colonic 5-HT levels and produced a concomitant reduction in arterial blood pressure relative to SHR→SHR controls (Figure S2B through S2D). Moreover, the transplant normalized the expression levels of tryptophan hydroxylase 1 (Tph1), the rate-limiting enzyme for 5-HT synthesis in enteroendocrine cells,^25^ in the colons of SHR recipients of FMT from WKY (Figure S2E). Conversely, FMT from SHR donors significantly reduced expression of Tph1 in the colon of WKY recipients (Figure S3C).

Because FMT of hypertensive microbiota reduced colonic 5-HT without altering luminal Trp, we next asked whether dysbiosis may suppress host 5-HT production through epigenetic mechanisms. To address this, we examined histone H3 lysine 9 acetylation, a chromatin modification strongly associated with active gene transcription,^27,28^ at the *Tph1* promoter. Colonic tissue from naïve WKY donors exhibited significantly higher H3 lysine 9 acetylation enrichment at the *Tph1* promoter compared with SHR donors (Figure S3A), indicating reduced transcriptional accessibility of the Tph1 locus in hypertensive rodents. Strikingly, WKY rats receiving FMT from SHR donors showed a significant reduction in H3 lysine 9 acetylation at the Tph1 promoter relative to WKY→WKY controls (Figure S3B), demonstrating that hypertensive microbiota is sufficient to epigenetically alter host colonic Tph1, consistent with reduced expression of colonic Tph1 in SHR→WKY recipients and its SHR donors (Figure S3C). In contrast, acetylation at the promoter of Tph2, the neuronal isoform of tryptophan hydroxylase, was low and unaffected by FMT (Figure S3A and S3B), indicating specificity for the Tph1-driven serotonergic pathway in the colon.

These findings demonstrate that impaired colonic serotonergic signaling is not only transmissible with hypertensive microbiota but is also reversible through microbial replacement. Together, these data identify gut microbiota as a reversible epigenetic regulator of host serotonergic signaling and blood pressure.

### Reduced Colonic Serotonin in Humans With Diagnosed Hypertension

Although dysregulated Trp metabolism has been described in humans with hypertension,^29–32^ whether intestinal serotonin production and serotonergic signal detection are altered at the level of the human gut has not been directly examined. To address this gap, we quantified colonic 5-HT levels and assessed expression of Tph1 and the 5HT3a receptor in colonic biopsy samples obtained from a small, age-matched cohort of hypertensive and normotensive individuals. Strikingly, individuals with diagnosed hypertension exhibited significantly reduced colonic 5-HT compared with normotensive controls (Figure 2), consistent with our rodent findings. This reduction was accompanied by lower expression of Tph1 and 5HT3a receptor expression in colonic tissue (Figure 2). Thus, human hypertension is associated with coordinated impairments in colonic serotonin production and serotonergic signal detection that closely mirror the molecular phenotype induced by hypertensive microbiota in rodents. These findings provide the first direct evidence that disruption of colonic serotonergic signaling is a conserved feature of hypertension across species and offers strong translational support for a gut-derived serotonergic pathway in blood pressure regulation.

### A Pivotal Role for Vagal Serotonin Signaling in Blood Pressure Homeostasis

Having established that the gut microbiota acts as a reversible epigenetic regulator of colonic serotonergic signaling and that this pathway is disrupted in both rodent models and humans with hypertension, we next tested whether serotonergic vagal sensory neurons are causally involved in blood pressure control. Although 5-HT is known to influence vascular tone,^21^ whether intestinal 5-HT can regulate blood pressure through defined neural circuits has not been directly tested. Intestinal 5-HT signaling is conveyed in part by the 5HT3a receptor expressed by a subset of vagal sensory neurons in the NG (NG^5HT3aR^).^33–37^ We first investigated the relative expression levels of these receptors along the vagal gut–brain axis and observed their decrease in NG, colon and the nucleus tractus solitarius (NTS) of WKY rats receiving FMT from the SHR (Figure S1D), whereas reverse transplant from WKY to SHR elevated the expression levels of 5HT3a receptors in the NG, colon and NTS of SHR rats receiving FMT from the WKY (Figure S2E). To determine whether the vagal afferent neurons expressing 5HT3a receptors are sufficient to acutely regulate cardiovascular function, we selectively activated NG^5HT3aR^ neurons using optogenetics while monitoring blood pressure and heart rate. To this end, we expressed the light-sensitive ion channel channelrhodopsin-2 (ChR2) in the NG^5HT3aR^ neurons of 5HT3aR^Cre^ (LE [Long-Evans]-Htr3a-^*em1(T2A-Cre)Sage*^) rats (Figure 3A). Bilateral NG injections of Cre-dependent adeno-associated virus (AAV) encoding ChR2 (AAV-DIO [double-floxed inverted open reading frame]-ChR2) were performed, whereas control rats received a Cre-dependent fluorophore not carrying ChR2 (AAV-DIO-tdTomato, see Extended Data for details on viral vectors; Figure 3A and 3D). Robust viral expression was confirmed in the NG cell bodies, their central projections within the NTS, and peripheral terminals in the proximal colon (Figure 3D; Figure S4A), consistent with targeting of gut-projecting vagal sensory neurons. Specificity of the Cre-dependent AAV for labeling the NG^5HT3aR^ neurons in 5HT3aR^Cre^ rats was confirmed by lack of viral gene expression in naïve Long-Evans rats injected with the Cre-dependent control virus (Figure S4B).

Bilateral optogenetic stimulation of NG^5HT3aR^ neurons in anesthetized rats by shining the excitatory blue light on the left and right NG separately^38–40^ evoked an immediate and time-locked increase in vagal afferent firing in ChR2-expressing animals (Figure 3B). This activation produced a pronounced and reversible decrease in blood pressure (≈20 mm Hg; Figure 3C and 3E) accompanied by a reduction in heart rate (≈40 beats per minute; Figure 3C and 3F). The effects typically persisted for the entire duration of light stimulation and slowly recovered to baseline after stimulus cessation (Figure 3C). In contrast, optical stimulation had no effect on vagal firing, blood pressure or heart rate in control animals lacking ChR2 expression (Figure 3A through 3F). These findings demonstrate that activation of NG^5HT3aR^ neurons is sufficient to acutely lower blood pressure and heart rate.

Because hypertension is a chronic condition, we next investigated whether the NG^5HT3aR^ neurons are required for long-term blood pressure homeostasis. To test necessity, we selectively ablated NG^5HT3aR^ neurons by bilaterally injecting a Cre-dependent AAV expressing Caspase 3 (AAV-DIO-taCasp3-TEVp) into the NG of 5HT3aR^Cre^ rats, with AAV-DIO-tdTomato serving as a control (Figure 3G; see Extended Data for details on viral vectors). Reverse transcription quantitative polymerase chain reaction (RT-qPCR) analysis confirmed that Caspase-mediated ablation significantly reduced 5HT3a receptor–expressing neurons in the NG (Figure 3H). Selective loss of NG^5HT3aR^ neurons produced a sustained progressive increase in blood pressure that emerged over several weeks and peaked 8 weeks after ablation compared with the Control group (Figure 3I). Heart rate was not significantly altered (Figure S4C), indicating a selective effect on blood pressure regulation. Together, these gain- and loss-of-function experiments establish NG^5HT3aR^ neurons as both sufficient and necessary for maintaining blood pressure homeostasis through serotonergic vagal sensory signaling.

### Blunted Vagal Sensitivity to Gut-Derived Serotonin in Hypertension

We next tested whether vagal sensory detection of 5-HT is functionally impaired in hypertensive rodents. RT-qPCR revealed significantly reduced 5HT3a receptor expression in SHR donors as well as the SHR→WKY FMT recipients, compared with WKY along the vagal gut–brain axis including the NG, colon, and NTS (Figure S1D). To determine whether reduced 5HT3a receptor expression translated to impaired functional sensing of serotonin, we examined vagal sensory neuron responses to administration of a specific 5HT3a receptor agonist, L-phenylbiguanide. To directly link neural activity with cardiovascular output, we combined in vivo calcium imaging of vagal sensory neurons with simultaneous telemetric blood pressure recording, enabling real-time correlation of individual neuronal responses with hemodynamic changes in the same animal (Figure 4A). For these experiments, we bilaterally injected AAV-hSyn-GCaMP6s (green fluorescent protein-calmodulin-M13 peptide sequence) into the NG of WKY and SHR rats to broadly express the calcium indicator GCaMP6 in vagal sensory neurons to serve as a proxy for neuronal activity. Two-photon microscopy was used to monitor neural activity in anesthetized animals with intact peripheral vagal connections, while increasing doses of L-phenylbiguanide were administered intravenously via the femoral vein to preferentially engage 5HT3a receptors (Figure 4; Figure S5). In normotensive WKY rats, L-phenylbiguanide elicited robust, dose-dependent activation of vagal sensory neurons, characterized by recruitment of an increased proportion of responsive neurons and larger calcium transients at higher doses (Figure 4B through 4D; Figure S5; Videos S1 through S3). These neuronal responses were time-locked to pronounced reductions in arterial blood pressure, with both neural and cardiovascular effects persisting for several minutes after cessation of infusion (Figure 4C and 4D).

In contrast, vagal sensory neurons from SHRs exhibited markedly blunted responses to serotonergic stimulation with L-phenylbiguanide. Across all tested doses, SHRs displayed a significantly reduced proportion of neurons as well as diminished calcium signaling amplitudes compared with WKY rats (Figure 4B through 4D; Figure S5; Videos S1 through S3). These attenuated neuronal responses were accompanied by smaller and less sustained reductions in blood pressure, indicating impaired serotonergic vagal control of cardiovascular function in hypertension (Figure 4C and 4D).

We next asked whether this sensory deficit was specifically localized to gut-projecting vagal sensory neurons. Colon-projecting neurons were selectively labeled by injecting a red-fluorescent retrograde neuronal tracer cholera toxin B into the submucosal wall of the proximal colons in WKY and SHR rats (Figure 4A and 4E). Two-color calcium imaging revealed that cholera toxin B–labeled, colon-projecting vagal sensory neurons from WKY rats responded robustly to L-phenylbiguanide, whereas responses in SHR were significantly diminished (Figure 4F and 4G; Figure S5). This deficit was evident both as a reduction in the fraction of cholera toxin B–positive neurons responding to serotonergic stimulation and as a decrease in response amplitude in individual neurons, indicating impairment of gut-innervating serotonergic vagal afferent signaling in hypertension.

Finally, because transplantation of hypertensive microbiota reduced colonic serotonin synthesis and suppressed 5HT3a receptor expression along the gut–brain vagal axis, we assessed whether serotonergic vagal control of blood pressure was similarly affected in the SHR→WKY recipients. Consistent with this prediction, the rapid and sustained reductions in blood pressure and heart rate elicited by L-phenylbiguanide in WKY→WKY controls were significantly blunted in SHR→WKY (Figure 4H and 4I), closely resembling the attenuated blood pressure responses observed in the WKY and SHR donors (Figure 4D). Together, these findings demonstrate that pro-hypertensive microbiota blunt serotonergic sensory signaling specifically within colon-innervating vagal sensory neurons, weakening neural-cardiovascular coupling and providing a mechanistic link between gut dysbiosis and hypertension.

### A Critical Role for Colonic Vagal Serotonin Signaling in Blood Pressure Homeostasis and Response to Stress

To determine whether serotonergic signaling from the colon is the relevant neural input controlling blood pressure, we next tested the causal role of colon-innervating vagal sensory neurons expressing 5HT3a receptors. Specifically, we investigated whether selective manipulation of this anatomically defined vagal subpopulation is: (1) sufficient to acutely regulate blood pressure, (2) necessary for long-term blood pressure homeostasis; and (3) relevant for cardiovascular responses to stress.

To determine whether this subpopulation is sufficient to acutely regulate blood pressure, we selectively stimulated colonic-projecting NG^5HT3aR^ neurons using optogenetics. A Cre-dependent retrograde virus expressing ChR2 (retrograde adeno-associated virus [AAVrg]-DIO-ChR2; see Extended Data for details on viral vectors) was injected in the wall of the proximal colon, whereas control animals received a retrograde virus expressing the tdTomato alone (Figure 5A). We observed ample labeling of the NTS, NG and colonic neuronal projections in 5HT3aR^Cre^ rats (Figure S6A) confirming viral gene expression. Bilateral optical stimulation of the NG in ChR2-expressing animals produced a rapid and robust reduction in blood pressure (Figure 5B and 5C), with an amplitude comparable to that observed after broad stimulation of all NG^5HT3aR^ neurons (Figure 3C and 3E). In contrast, heart rate responses to selective stimulation of colon-innervating NG^5HT3aR^ neurons were markedly smaller than those observed after broad stimulation of NG^5HT3aR^ neurons (Figure 3C and 3F), suggesting that regulation of heart rate involves additional 5HT3a receptor-expressing vagal populations that do not innervate the colon. Control animals exhibited no changes in blood pressure or heart rate in response to optical stimulation (Figure 5B and 5D). These findings demonstrate that activation of colon-innervating serotonergic vagal sensory neurons is sufficient to acutely lower blood pressure.

We next tested whether colon-innervating NG^5HT3aR^ neurons are required for long-term blood pressure homeostasis. To selectively ablate this population, we utilized an intersectional genetic strategy, in which 5HT3aR^Cre^ rats received colonic injections of Cre-dependent retrograde virus expressing FLPo (Flp recombinase, optimized) recombinase (AAVrg-DIO-FLPo; see Extended Data for details on viral vectors; Figure 5E), followed by bilateral NG injections of a new FLPo-dependent viral construct expressing caspase 3 (AAV-fDIO-taCasp3-TEVp; see Extended Data for details on viral vectors; Figure 5E). Control animals received the same viral strategy but expressed a fluorescent reporter instead of caspase (Figure 5E). This approach resulted in selective targeting of colon-innervating NG^5HT3aR^ neurons (Figure S6B) and the caspase-containing virus produced a significant reduction in 5HT3a receptor expression in NG compared with controls (Figure 5G). Selective ablation of colonic NG^5HT3aR^ neurons led to a progressive, time-dependent increase in blood pressure, which peaked 8 weeks after viral transfection, compared with control animals (Figure 5F; Figure S6C). Heart rate was not significantly affected (Figure S6C, far right), indicating a selective role for colonic serotonergic vagal signaling in blood pressure regulation rather than generalized autonomic disruption. In addition, we observed no differences in overall gut microbiota composition and abundances in the 2 groups (Figure S6D), confirming specificity of neural effects. These findings establish that intact colonic NG^5HT3aR^ signaling is necessary for long-term maintenance of blood pressure homeostasis.

Because stress is intricately linked to hypertension and gut dysbiosis^41^ and because vagal signaling is known to modulate stress responses,^42^ we next asked whether loss of colonic NG^5HT3aR^ neurons exacerbates stress-induced elevations in blood pressure. Acute restraint produced a significantly greater increase in arterial blood pressure in rats with caspase-ablated colonic NG^5HT3aR^ neurons compared with controls (Figure 5H), indicating heightened sensitivity to stress. To further probe this interaction, we infused angiotensin II (ANGII), a key pro-hypertensive hormone closely associated with stress responses.^43^ Continuous infusion of ANGII for one week produced a greater elevation in blood pressure in rats lacking colonic NG^5HT3aR^ neurons than in control rats (Figure 5I), demonstrating that loss of intestinal serotonergic vagal signaling enhances vulnerability to both psychological and hormonal stressors that affect blood pressure. Together, these gain- and loss-of-function experiments identify colon-innervating 5HT3a receptor–expressing vagal sensory neurons as a critical neural substrate for blood pressure homeostasis and reveal a previously unrecognized role for intestinal serotonergic vagal signaling in buffering stress-induced hypertension.

### Recovery of Vagal Serotonergic Signaling in the Colon Can Alleviate Dysbiosis-Induced Hypertension

Because hypertensive microbiota suppresses colonic serotonergic signaling and reduces 5HT3a receptor expression along the gut–brain vagal axis, and because reversing the dysbiosis is sufficient to decrease blood pressure and restore colonic 5-HT and 5HT3a receptors, we next asked whether restoring the serotonergic vagal signaling selectively within colon-innervating NG^5HT3aR^ neurons could reverse established hypertension. To address this, we focused on hypertensive SHR/5HT3aR^Cre^ rats, which exhibit gut dysbiosis and impaired serotonergic vagal signaling (Figure S7A and S7B). Using an intersectional viral strategy, we selectively increased 5HT3a receptor expression in colon-innervating NG^5HT3aR^ neurons (Figure 6A and 6C). A Cre-dependent retrograde virus expressing FLPo recombinase was injected into the wall of the proximal colon, followed by bilateral NG injection of a FLPo-dependent virus encoding the 5HT3a receptor (AAV-fDIO-5HT3aR; Figure 6A). Control rats received an identical strategy but expressed a fluorescent reporter (AAV-fDIO-YFP [yellow fluorescent protein]) instead (Figure 6A). This approach resulted in selective viral labeling of colon-projecting NG^5HT3aR^ neurons in both groups (Figure 6B) and produced an approximately 30% increase in 5HT3a receptor expression in the NG of rats receiving the 5HT3a receptor–expressing virus, as confirmed by RT-qPCR (Figure 6C).

Remarkably, selective restoration of 5HT3a receptor expression in colon-projecting vagal sensory neurons attenuated the progressive development of hypertension in SHR/5HT3aR^Cre^ rats (Figure 6D; Figure S7C). This antihypertensive effect was further enhanced by dietary supplementation with tryptophan (5% Trp), which increased colonic 5-HT levels similarly in both groups (Figure 6E) compared with naïve SHR, without altering gut microbial community structure or diversity (Figure 6F). These findings demonstrate that colonic serotonergic vagal signaling, through combined restoration of receptor expression and ligand availability, is sufficient to mitigate dysbiosis-associated hypertension independently of microbiota remodeling. Together, these results identify colonic 5HT3a receptor–dependent vagal signaling as a therapeutically actionable node linking gut dysbiosis to hypertension and demonstrate that targeted restoration of this pathway can reverse disease progression.

## DISCUSSION

Our study identifies a previously unrecognized role of intestinal vagal sensory neurons in orchestrating the communication between the gut microbiota and the cardiovascular system to maintain the blood pressure homeostasis. We delineate a distinct population of colon-innervating vagal sensory neurons responsive to 5-HT whose activity is reduced in hypertension, and whose function is shaped by gut microbiota. Although previous studies have reported associations between gut dysbiosis and elevated blood pressure in rodents and humans,^7–9,17^ the mechanisms by which microbial signals are sensed by the host and translated into sustained changes in blood pressure regulation have remained unclear. Our findings address this gap by defining a gut-derived sensory pathway that causally links microbial state to neurogenic control of blood pressure.

Conceptually, these results extend the emerging framework of “neurobiotic sensing,” a term previously introduced to describe neural pathways that enable the host to detect and respond to molecular cues derived from resident microbes.^44^ In that work, microbial products were shown to communicate via enteroendocrine cell release of PYY (peptide YY) to recruit a neural circuit that shapes host eating behavior. Here, we expand this concept to cardiovascular physiology by identifying a serotonergic neurobiotic pathway through which microbiota-dependent signals from the colon epigenetically alter 5-HT production detected by vagal afferent neurons to integrate into blood pressure control. Together, these studies support the broader idea that the gut is not merely a metabolic or immune interface, but also a sensory organ capable of shaping systemic physiology through dedicated neural circuits. The vagus nerve, a master regulator of homeostatic physiology and a principal conduit for gut–brain communication,^45^ plays a direct and essential role in blood pressure regulation via serotonergic signaling. In doing so, our work integrates several previously disconnected observations: (1) the influence of the gut microbiota on intestinal serotonin production,^46–48^ (2) alterations in tryptophan metabolism in rodent and human hypertension,^21–23,29–32^ and (3) the contribution of vagal pathways to neurogenic hypertension. By linking these processes mechanistically, we identify a neural pathway through which microbial modulation of intestinal serotonin directly impacts cardiovascular function.

Recent advances in the cellular mapping of enterochromaffin cells add important context to our findings. A comprehensive single-cell transcriptomic and imaging analysis has revealed that enterochromaffin cells in the gut are not a uniform population but rather consist of multiple distinct subtypes with different sensory and signaling properties.^49^ This molecular and spatial diversity suggests that enterochromaffin subsets are specialized in detecting luminal or microbial cues. Our work complements these insights by showing that those enterochromaffin cells that release 5-HT in response to microbial influences engage a defined vagal sensory circuit that affects blood pressure. In this framework, shifts in the gut microbial community, potentially driven by environmental or dietary factors, could preferentially alter the activity of the enterochromaffin subtypes most tightly coupled to cardiovascular control, thereby contributing to dysbiosis-associated hypertension and stress-responsive blood pressure changes.

Using intersectional genetic strategies and new viral tools, we further demonstrate that 5-HT–sensing colonic vagal neurons are both sufficient and necessary for blood pressure regulation. Selective activation of these neurons acutely lowers blood pressure, whereas their disruption produces progressive hypertension and exaggerated cardiovascular responses to stress. Given the distributed and partially redundant nature of cardiovascular control, it is notable that manipulation of this single molecularly and anatomically defined vagal sensory population produced sustained changes in blood pressure of ≈10–20 mm Hg. These findings shift the prevailing view of hypertension away from a model dominated solely by cardiac, renal, or central mechanisms and toward one that incorporates peripheral sensory inputs from the gut as active drivers of hypertension.

Although the precise central networks downstream of these vagal sensory neurons remain incompletely defined, previous studies implicate discrete neuronal populations within the NTS in processing serotonergic vagal input and coordinating feeding, stress and autonomic responses.^50–55^ The convergence of these functions within brainstem circuits suggests a shared neural architecture through which intestinal signals influence multiple aspects of physiological state, including cardiovascular regulation. A notable technical aspect of this work is the ability to simultaneously record activity from identified vagal sensory neurons while monitoring arterial blood pressure in real time, providing a direct functional link between gut-evoked neural signals and cardiovascular output. This approach offers a framework for dissecting how peripheral sensory inputs are dynamically integrated into central autonomic control and how these processes are remodeled in disease.

The overlap between feeding related gut signaling and blood pressure regulation may be particularly relevant, as intestinal 5-HT released in response to nutrient ingestion^56^ not only promotes digestion, secretion, and motility,^25^ but can also reduce sympathetic nervous system activity and promote intestinal vasodilation and postprandial hyperemia.^57,58^ Consistent with this idea, GI dysfunction and diminished intestinal blood flow have been reported in the SHR,^8^ whereas constipation has been documented in subsets of hypertensive patients,^59,60^ further linking GI pathology with hypertension. Together, these observations support the view that serotonergic gut-vagal signaling coordinates postprandial and cardiovascular physiology, and that disruption of this integration may contribute to disease. Dissecting how these pathways are integrated and remodeled in disease will be an important direction for future research.

We also observed a marked potentiation of stress-induced hypertensive responses after disruption of intestinal serotonergic vagal signaling. This aligns with emerging evidence linking microbiota–vagal axis and Trp metabolism in conditions of stress^61^ and with findings of exaggerated blood pressure responses to pro-hypertensive microbiota transfer under restraint.^17,19^ Together, these findings suggest that serotonergic neurobiotic signaling from the gut not only contributes to basal blood pressure homeostasis but also buffers cardiovascular responses to daily psychological and hormonal stressors.

At the molecular level, reduced expression of colonic Tph1 after transplantation of pro-hypertensive microbiota correlated with reduced colonic 5-HT levels, despite no change in either the colonic or bacterial Trp or bacterial 5-HT. This indicates that reduced intestinal 5-HT in hypertension reflects direct microbial modulation of host serotonergic pathways rather than altered substrate availability. Our findings suggest that this occurs, at least in part, through epigenetic repression of Tph1, as hypertensive microbiota reduced H3 lysine 9 acetylation enrichment at the Tph1 promoter. Gut bacteria are known to regulate intestinal 5-HT synthesis through metabolites including butyrate,^62,63^ but how this may align with our model remains to be tested directly. Because gut microbial composition and metabolite production are strongly influenced by external factors such as diet and antibiotic exposure, dietary interventions that account for the modern food environment characterized by widespread consumption of Westernized diets,^64^ together with targeted prebiotic and probiotic strategies^65^ may represent viable adjunctive approaches to restore intestinal serotonergic signaling in combination with existing antihypertensive therapies.

Importantly, the translational relevance of this neurobiotic pathway is supported by our observation that individuals with diagnosed hypertension exhibit reduced colonic 5-HT levels together with decreased expression of Tph1 and 5HT3a receptor in colonic biopsies. These findings suggest that impaired intestinal serotonergic signaling is conserved across species and identify the gut serotonergic axis as a potential clinically relevant regulator of blood pressure. Resistant hypertension, defined by unresponsiveness to ≥3 antihypertensive medications,^66^ affects up to 20% of hypertensive patients,^4–6,67^ and current therapies primarily target cardiovascular, neural, and renal mechanisms. Such approaches may therefore be insufficient to address hypertension driven by altered microbiota-gut–brain signaling. By identifying serotonergic neurobiotic signaling as a peripherally accessible and reversible regulator of blood pressure, our findings reveal a previously unrecognized mechanism through which the gut microbiota engages vagal sensory pathways to shape cardiovascular homeostasis. Our work points to new therapeutic strategies that combine neural, dietary, and microbiota-targeted interventions for the treatment of hypertension marked by gut dysbiosis.

### Limitations

This study has several limitations. First, although we provide mechanistic and causal evidence in rodent models, the human data are limited in scale and observational in nature. Although the reduced colonic serotonergic signaling observed in individuals with diagnosed hypertension supports the translational relevance of our findings, larger and longitudinal human studies will be required to establish causality and determine the extent to which this pathway contributes to blood pressure regulation across diverse patient populations. Because this validation cohort was small (N=5 per group) and predominantly female (83%), the human findings should be interpreted as confirmatory of the rodent mechanism rather than as generalizable estimates of effect across the broader hypertensive population; larger and more demographically diverse cohorts are needed. Second, our work focuses on a defined serotonergic vagal sensory pathway originating in the colon; however, additional gut-derived signals and neural circuits are likely to contribute to microbiota-dependent regulation of blood pressure and were not examined here. Third, although we identify epigenetic regulation of *Tph1* as a mechanism linking hypertensive microbiota to reduced intestinal 5-HT, the specific microbial species and metabolites responsible for this effect remain to be fully elucidated. Finally, our experiments were conducted in controlled experimental settings, and how dietary patterns, environmental factors, or chronic disease states interact with this neurobiotic pathway in humans will require further investigation. Despite these limitations, the convergence of microbiota manipulation, neural circuit dissection, and translational human data provides strong support for a previously unrecognized gut-vagal mechanism in blood pressure regulation.

## ARTICLE INFORMATION

### Acknowledgments

The authors would like to thank Allen Schroering at the University of Toledo Integrated Core Facilities for technical support with immunohistochemistry. The authors also thank Niousha Ahmari for assisting with reverse transcription quantitative polymerase chain reaction analyses in nodose ganglia.

### Author Contributions

A.d.A., E.W.D., H.S.K., and T.Y. conducted various experiments, performed data analyses, and edited the manuscript. H.S.K. and A.A.P. performed bacterial sequencing analyses and 5-HT assays. E.B.O. performed tissue analyses in human colonic samples, created the graphical abstract, and edited the manuscript. N.R.B. and E.B.O. performed animal husbandry, tissue collections, and conducted reverse transcription quantitative polymerase chain reaction experiments and analyses. B.D.A. performed FMT experiments and blood pressure analyses. D.M.B. performed blood pressure measurements during in vivo calcium imaging and edited the manuscript. T.J.G. and C.J.M. performed metabolomics experiments and analyses and edited the manuscript. D.S.M. performed tissue cutting, immunohistochemistry, and imaging. A.S. performed blood pressure measurements and edited the manuscript. A.L.K. and H.S.K. performed confocal imaging and edited the manuscript. M.R.D. and I.d.l.S. performed epigenetic experiments. B.S. and M.L.A. provided colonic tissue samples. G.d.L. and J.Z. conducted the experiments, performed the data analyses, provided funding, and wrote and edited the manuscript. All authors approved the final version of the manuscript.

### Disclosures

J.Z. is a co-founder of and holds an equity/ownership interest in Panthea Inc, which is developing microbiome- and postbiotic-based products related to the intestinal serotonergic pathway described in this work. The remaining authors declare no competing interests.

### Supplemental Material

Expanded Methods

Figures S1–S7

Tables S1–S7

Videos S1–S3

Excel Files (Tables S4–S7)

References 68–83

## Supplementary Material
