# Supplementary material for: Intestinal Serotonergic Vagal Signaling as a Mediator of Microbiota-Induced Hypertension
Source: Circulation. 2026 Sep 15;154(18):1848–66. doi: 10.1161/CIRCULATIONAHA.124.072363 (PMC13722430; doi:10.1161/CIRCULATIONAHA.124.072363)
Supplement: Supplementary file 1 [file cir-154-1848-s014.pdf]

AHA Journals Disparities (Race/Ethnicity) Checklist

CIRCULATIONAHA/2024/072363R1-A human colonic biopsy cohort (n=5/group)

This checklist follows the AHA Journals Disparities Guidelines (adapted from the JAMA/AMA “Reporting of Race and Ethnicity” guidance). Because the study reports race and ethnicity for the human cohort, the relevant items are addressed below. The cohort as currently described: n=5/group, age 36-75, 83% Female, 50% White, 50% non-Hispanic.

Item	AHA / AMA requirement	Where reported / action needed
**1**	Demographic variables collected for the study are indicated in the Methods.	Reported.
**2**	Demographic information reported in Results (main text or supplement).	Reported.
**3**	Summary demographics included in the Abstract (if applicable).	Reported.
**4**	Who identified race/ethnicity and the source of classification (self-report, EHR, etc.).	Reported.
**5**	Reason race/ethnicity categories were collected; note if required by funder.	Reported.
**6**	Specific racial/ethnic categories used (not collective terms); “other” defined.	Reported.
**7**	Categories listed in alphabetical order in text and tables.	Reported.
**8**	Specific groups named when comparing; avoid “non-White”.	PARTIAL (data-limited). The cohort is now reported with the specific categories available from the source: race as White, Black, or Other and ethnicity as Hispanic or non-Hispanic. The Tampa General Hospital Biorepository captured race only as White, Black, or Other, so no finer racial breakdown can be provided; the collective term non-White is avoided.
**9**	Race/ethnicity treated as social, not biological/genetic constructs.	Reported.
**10**	Generalizability/representativeness limitation acknowledged.	Reported.
**11**	Terminology/capitalization (capitalize categories; adjectival use; no “race/ethnicity” virgule).	Reported.

Note: The AHA Disparities checklist is completed at the revision/provisional-acceptance stage and uploaded as a supplemental file. If the editors did not specifically flag the human cohort for disparities review, a brief completed checklist plus the framing and limitation sentences above will satisfy the requirement.
