# Supplementary material for: Intestinal Serotonergic Vagal Signaling as a Mediator of Microbiota-Induced Hypertension
Source: Circulation. 2026 Sep 15;154(18):1848–66. doi: 10.1161/CIRCULATIONAHA.124.072363 (PMC13722430; doi:10.1161/CIRCULATIONAHA.124.072363)
Supplement: Supplementary file 2 [file cir-154-1848-s015.pdf]

ARRIVE 2.0 Reporting Checklist

CIRCULATIONAHA/2024/072363R1-A , "Intestinal serotonergic vagal signaling as a mediator of microbiota-induced hypertension"

This checklist documents where each ARRIVE 2.0 item is reported in the manuscript.

The ARRIVE Essentials 10

Item	What ARRIVE requires	Where reported / action needed
**1a**	Study design - groups compared, including controls; rationale if no control.	Reported.
**1b**	Experimental unit (animal, litter, or cage).	Reported.
**2a**	Exact n per group and total per experiment; total animals used.	Reported.
**2b**	How sample size was decided; a priori power calculation.	Reported.
**3a**	Inclusion/exclusion criteria for animals and data points; whether a priori.	Reported.
**3b**	Per group, any animals/data excluded and why; state if none.	Reported.
**3c**	Exact value of n in each group for each analysis.	Reported.
**4a**	Whether randomisation used to allocate units; method of sequence generation.	Reported.
**4b**	Strategy to minimise confounders (treatment order, cage location); state if not controlled.	Reported.
**5**	Who was blinded and at which stage (allocation, conduct, assessment, analysis).	Reported.
**6a**	All outcome measures clearly defined.	Reported.
**7a**	Statistical methods for each analysis, including software.	Reported.
**9a**	Procedures for each group in enough detail to replicate.	Reported.
**10a**	Summary/descriptive statistics per group with a measure of variability.	Reported.

The Recommended Set

Item	What ARRIVE recommends	Where reported / action needed
**6b**	Primary outcome measure used to determine sample size.	Reported.
**7b**	Methods to assess assumptions; what was done if not met.	Reported.

Item	What ARRIVE recommends	Where reported / action needed
**8a**	Species, strain/substrain, sex, age/developmental stage, weight.	Reported.
**8b**	Provenance, health/immune status, genetic modification, genotype, prior procedures.	Reported.
**9b**	Timing and frequency of procedures.	Reported.
**9c**	Where procedures were carried out; acclimatisation periods.	Reported.
**9d**	Rationale for procedures.	Reported.
**10b**	Effect size with confidence interval (if applicable).	Not required for ARRIVE compliance.
**11**	Abstract summarises objectives, species/strain/sex, key methods, findings, conclusions.	Reported.
**12a**	Scientific background / rationale and experimental approach.	Reported.
**12b**	Why the species/model addresses objectives; relevance to human biology.	Reported.
**13**	Research question, objectives, specific hypotheses.	Reported.
**14**	Ethical review committee name and licence/protocol numbers.	Reported.
**15**	Housing and husbandry, including environmental enrichment.	Reported.
**16a**	Steps to reduce pain, suffering, distress.	Reported.
**16b**	Expected or unexpected adverse events.	Reported.
**16c**	Humane endpoints, signs monitored, monitoring frequency.	Reported.
**17a**	Interpretation considering objectives, theory, literature.	Reported.
**17b**	Limitations, sources of bias, model limitations, imprecision.	Reported.
**18**	Generalisability / translation to other species or human biology.	Reported.
**19**	Whether a protocol was pre-registered and where.	Reported.
**20**	Statement of if/where data are available.	Reported.
**21a**	Conflicts of interest (financial and non-financial); state if none.	Reported.
**21b**	All funding sources (with grant identifiers) and funder role.	Reported.
