# Supplementary material for: Intestinal Serotonergic Vagal Signaling as a Mediator of Microbiota-Induced Hypertension
Source: Circulation. 2026 Sep 15;154(18):1848–66. doi: 10.1161/CIRCULATIONAHA.124.072363 (PMC13722430; doi:10.1161/CIRCULATIONAHA.124.072363)
Supplement: Supplementary file 3 [file cir-154-1848-s001.docx]

**Methods**

Detailed experimental procedures are provided below.

**Data Availability**

16S sequencing data is available at PRJNA1140871 [http://www.ncbi.nlm.nih.gov/bioproject/1140871](https://nam04.safelinks.protection.outlook.com/?url=http%3A%2F%2Fwww.ncbi.nlm.nih.gov%2Fbioproject%2F1140871&data=05%7C02%7CJasenka.Zubcevic%40UToledo.Edu%7C824cbc75aa2d45647bc208dcae6d26cc%7C1d6b1707baa94a3da8f8deabfb3d467b%7C0%7C0%7C638577031037989826%7CUnknown%7CTWFpbGZsb3d8eyJWIjoiMC4wLjAwMDAiLCJQIjoiV2luMzIiLCJBTiI6Ik1haWwiLCJXVCI6Mn0%3D%7C0%7C%7C%7C&sdata=TwsK5YNVrZ24ERvDVbIZPmdKwEbWQViKFrxCflRYiaQ%3D&reserved=0).Untargeted and targeted metabolomics data are deposited at the NIH Common Fund Metabolomics Workbench (Metabolomics Data Repository and Coordinating Center; www.metabolomicsworkbench.org) under REQ20260618220704. Normalized metabolite measures are provided in Table S7. Additional data and code are available upon request to the corresponding authors.

**Experimental rat models**

All procedures were approved by the Institutional Animal Care and Use Committee at the University of South Florida, University of Toledo, and University of Florida. Studies were performed in male and female rats aged 2-5 months. Rats were group housed until telemetry surgery in a specific pathogen-free facility (22ºC, 65% humidity and 12h light:dark cycle) with free access to regular chow and systems water. Animals were housed with environmental enrichment and one animal per cage. Animals were randomly assigned to experimental groups by cage. The experimental unit was the individual animal. Inclusion and exclusion criteria were defined a priori: animals were excluded only for protocol-defined humane endpoints, failed surgery, or confirmed off-target viral expression. Investigators performing blood pressure telemetry analysis, calcium-imaging quantification, and histological scoring were blinded to group allocation; surgical procedures and tissue collection could not always be blinded owing to the visible nature of the intervention. For all survival surgeries (telemetry probe implantation, viral vector and nodose ganglion injections), anesthesia was induced and maintained with isoflurane (4% at induction in 95/5% O_2_/CO_2,_ maintained at 1.5-2% throughout) and buprenorphine was used post-operatively at an IACUC approved dose. Animals were allowed approximately 1 week of post-surgical recovery before baseline recording and approximately 2 hours of acclimatization to the telemetry recording room before each session. Telemetry recordings were started at a consistent time of day and recorded continuously for 24-48hrs, and treatment groups were interleaved across cages. Animals were monitored daily; predefined humane endpoints (>20% body-weight loss, signs of distress, or post-surgical complications) prompted veterinary review and euthanasia per IACUC protocol. No unexpected adverse events were observed. Strains used were as follows: male WKY (normotensive, Charles River), male and female SHR (hypertensive, Charles River), and male and female HsdSage:LE-*5ht3a*^em1(T2-Cre)Sage^ (normotensive, 5HT3aR^Cre^, Inotiv). To generate SHR/5HT3aR^Cre^, female SHR was cross bred with male 5HT3aR^Cre^. FMT experiments were performed on male WKY and SHR (by oral gavage, as per [18]) and on male and female SHR/5HT3aR^Cre^ offspring by biweekly transfer of feces in bedding from adult sex matched SHR donors [68] starting at weaning until 3 months of age. Success of FMT was confirmed by 16S rRNA sequencing and analysis [10].

**Metabolomic analyses in colons and feces**

Samples were homogenized at 50 mg/mL in 5 mM Ammonium acetate. Protein concentration of colon samples was normalized to 1100 ug/mL, and fecal samples were normalized per g of wet feces. Global metabolomics profiling was performed on a Thermo Q-Exactive Orbitrap mass spectrometer with Dionex UHPLC. Samples were analyzed in positive and negative heated electrospray ionization with a mass resolution of 35,000 at m/z 200 as separate injections. Separation was achieved on an ACE 18-pfp 100 x 2.1 mm, 2 µm column with mobile phase A as 0.1% formic acid in water and mobile phase B as acetonitrile. The column flow rate was 350 µL/min at 25°C. 4 µL was injected for negative and 2 µL for positive ions. MZmine (freeware) was used to identify features, deisotope, and align features. All adducts and complexes were identified and removed from the data set. The mass and retention time data was searched against our internal metabolite library [69, 70]. Inner-quartile range filtering was implemented in the R package MetaboAnalystR (<https://github.com/xia-lab/MetaboAnalystR>). Missing data were imputed by k-nearest neighbor imputation in the R package MetaboAnalystR. Peak intensities were normalized sample-wise using sum normalization followed by log (base 10) transformation and Pareto scaling in R package MetaboAnalystR. Multivariate and univariate statistical analysis assessed clustering of samples based on metabolomic profiles to identify metabolites with changes in abundance between groups or biological conditions. The list of compounds detected in positive and negative ionization modes was combined and reduced to a single representative compound per likely metabolite based on p-value (lowest p-value compound retained). For targeted tryptophan metabolomics, the target analytes, L-tryptophan and 5-HT were obtained from Sigma (St Louis, MO), L-kynurenine, kynurenic acid and xanthurenic acid were obtained from MP biomedicals (Santa Ana, CA), and anthranilic acid was obtained from Fluka (Charlotte, NC). L-tryptophan 13C11 and L-kynurenine sulfate:H2O (ring D4) were obtained from Cambridge Isotopes (Tewksbury, MA), serotonin-D4, kynurenic acid-D5 and xanthurenic acid-D4 were obtained from Santa Cruz (Dallas, TX), and anthranilic acid-13C6 and xanthurenic acid-D4 were obtained from ISOTEC (St Louis, MA). All other reagents used were of LC-MS purity and obtained from ThermoScientific. Bovine serum albumin (BSA; Sigma) was prepared to a concentration of 70 g/L in LC-MS grade water. A stock calibration mix was prepared in BSA with Trp at 80,000 ng/mL, 5-HT and kynurenine at 8,000 ng/mL, kynurenic acid, xanthurenic acid and anthranilic acid at 4,000 ng/mL. A 9-point calibration curve was created with serial dilution covering 100 – 25,600 ng/mL for Trp, 10 – 2,560 ng/mL for 5-HT and kynurenine, and 5 – 1,280 for kynurenic acid, xanthurenic acid and anthranilic acid. The internal standard solution was prepared with Trp-13C11, 5-HT-D4 and kynurenine-D4 at 5 µg/mL, kynurenic acid-D5 at 0.5 µg/mL and anthranilic acid 13C6 and xanthurenic acid-D4 at 0.8 µg/mL in 90:10 water:methanol. Sample analysis was conducted on a ThermoScientific Q-Exactive Orbitrap with Dionex 3000 UHPLC. Briefly, samples were analyzed in positive heated electrospray ionization with the following source parameters: spray voltage = 3500 V, aux gas = 10, sheath gas = 50, capillary temperature = 325 °C, spray gas = 1, and S-lens RF level = 30. Separation was achieved on an ACE 18-PFP 100 x 2.1 mm, 2 µm column (Mac-Mod Analytical, Chadds Ford, PA) using a gradient with mobile phase A as 0.1% formic acid in water and mobile phase B as acetonitrile with a column temperature of 25°C. Gradient elution was ramped from 0% B to 80% B over 13.0 min at 350 µL/min and increased to 600 µL/min from 16.80 and 17.50 min for re-equilibration. The runtime was 20.50 min and full scan at 35,000 mass resolution was acquired from 2 µL injection in positive ion mode. Calibration was conducted by extraction of the exact mass for each target analyte and reference to the respective internal standard.

**Colonic samples from Tampa General Hospital Biorepository**

Human colonic mucosa tissue samples were obtained from University of South Florida/Tampa General Hospital Precision Medicine Biorepository with Internal Review Board approval (STUDY000356). Biopsy samples (~0.5 cm^3^) from mucosa with no endoscopically visible malignancy were obtained during routine colonoscopy with informed consent. Normotensive and hypertensive groups (n=5/grp, age 36-75, 83% Female) were defined based on hypertension diagnosis absence or presence in medical records, respectively. Participant race and ethnicity were self-reported by participants using fixed categories based on the U.S. Office of Management and Budget standards, as recorded in the Tampa General Hospital Precision Medicine Biorepository. Race and ethnicity are reported here as social, not biological, constructs and are provided solely to describe the demographic composition of the cohort. They were not used as biological variables and were not entered as covariates in any analysis. The human cohort comprised participants who self-identified as White, Black, or Other. Because this validation cohort was small (N=5 per group) and predominantly female (83%), the human findings should be interpreted as confirmatory of the rodent mechanism rather than as generalizable estimates of effect across the broader hypertensive population; larger and more demographically diverse cohorts are needed’.

**Serotonin (5-HT) assay in the colon**

5-HT ELISA was performed at endpoint in proximal colons of adult rats and in human colonic samples. Colons were dissected at endpoint and flash frozen. Homogenized tissues (300 µl of RIPA buffer with protease and phosphatase inhibitor cocktail, Abcam, Cambridge, UK) were used to measure 5-HT levels in technical triplicates using a competitive enzyme-linked immunosorbent assay (ELISA) following the manufacturer's instructions (Rocky Mountain Diagnostics, CO, USA, Cat#: BA E-5900R) and normalized for tissue protein.

**ChIP PCR assay in the proximal colon**

Frozen rat colons were pulverized into a fine powder using a mortar and pestle, followed by crosslinking with 1% formaldehyde at room temperature for 15 minutes. The crosslinked tissue was then homogenized in Lysis Buffer 1 (50mM HEPES-KOH, pH 7.5, 140mM NaCl, 1mM EDTA, 10% glycerol, 0.5% NP40, 0.25% Triton X, and protease inhibitors) [71]. Nuclei were washed with Lysis Buffer 2 (10mM Tris-HCl, pH 8.0, 200mM NaCl, 1mM EDTA, 0.5mM EGTA and protease inhibitors) and re-suspended in Lysis Buffer 3 (10mM Tris-HCl, pH 8.0. 100mM NaCl, 9mM EDTA, 0.5mM EGTA, 0.1% Na-Deoxycholate, and 0.5% N-lauryolsarcosine, protease inhibitors). The lysate was then sonicated in 30 second pulses at 60% power in 20S pulses for a total of 5 minutes to get an average fragment size of 200-1000 base pairs. A small aliquot (1%) of the chromatin was reserved as input control. Approximately 50μg of chromatin was pre-cleared for one hour at 4^o^C with Protein A Sepharose (Cytiva, Marlborough, MA, USA), then incubated overnight with an antibody to acetylated-histone H3 (Lys 9) (C5B11, Cell Signaling Technology, Danvers, MA, USA) or normal rabbit IgG (2729, Cell Signaling Technology, St. Louis, MO, USA) in Lysis Buffer 3, to which 1.1% Triton X-100 was added. Protein A sepharose was then added and incubation continued for an additional four hours. Samples were then washed five times in RIPA buffer (50 mM HEPES, 500 mM LiCl, 0.1 mM EDTA, 1.0% NP-40 and 0.7% Na-Deoxycholate) and once with TE containing 50mM NaCl. Bound chromatin was eluted in 50 mM Tris (pH 8.0), 10 mM EDTA, and 1.0% SDS. Crosslinks were reversed by overnight incubation at 65°C. After proteinase K digestion, DNA was purified by phenol/chloroform extraction and ethanol precipitation. Promoter regions of *Tph1* and *Tph2* were retrieved from the UCSC Genome Browser, and primers were designed using Primer3 (version 4.1.0). PCR amplifications were carried out on an ABI 7500 system (Thermo Fisher, Waltham, MA, USA) using SYBR green master mix (Qiagen, Hilden, Germany). ΔCT values were calculated by subtracting the CT values obtained from ChIP samples using the antibodies of interest from the CT values of the corresponding input controls. The 2-ΔΔCt method was then used to calculate enrichment of immunoprecipitated material, reported as percent input. Primer sequences for ChIPs: *Tph1*: *F: 5’-CCCATTGGCCATTCTGAGGA-3’ and R: 5’-CCACCCCACTCACCTCAAA -3’ and Tph2: 5’TGAGCACAGATAACCCCAGG-3’ and 5’-CCCCTCCCTGTGTTCCTTTA-3’*.

**Bacterial DNA extraction, 16S rRNA sequencing and data analysis**

Fecal and/or cecal samples were collected for 16S rRNA gene sequencing as per [10, 72, 73]. Briefly, bacterial DNA extraction was performed using QIAam®PowerFecal®DNA kit (QIAGEN) and bacterial 16S rRNA gene sequencing was performed on an Illumina MiSeq (Illumina, San Diego,CA) platform. Demultiplexed reads were analyzed using QIIME 2 [v.2023.2] [10, 73, 74]. DADA2 plugin was used to merge paired-end FastQ files, denoising, chimera removal and construction of amplicon sequence variants (ASV) [75]. Taxonomy was analyzed using the Silva v.132 databases at 99% identity pre-trained for the V3-V4 region [76]. Rarefaction was performed at a sampling depth of the sample with the minimum amount of reads per sample. Alpha-diversity was estimated using 3 metrics: Shannon, Observed features and Simpson. Beta-diversity was performed using the Bray Curtis dissimilarity index and visualized using principal coordinate analysis (PCoA). Permutational Multivariate Analysis of Variance (PERMANOVA, adonis, R, 999 permutations) was used to test whether differences between groups [10, 73, 74, 76, 77].

**General surgical procedures**

Radiotelemetry surgeries were performed as per [10, 14]. Briefly, rats were anaesthetized with isoflurane (induction at 4%; maintenance at 2% in 100% medical grade O_2_). Radiotelemetry transmitters (HD-S10, DSI) were implanted in the abdominal or femoral arteries for continuous collection of blood pressure and heart rate in conscious unrestrained rats. Following recovery, blood pressure and heart rate measurements were taken once weekly over 24hrs, averaged and presented as change (Δ) from baseline (i.e., week 0, Wk0) within experimental groups.

Viral vectors were delivered to the wall of the proximal colon and bilaterally in the NG as previously described [40, 78]. For NG injections, in anaesthetized rats, a midline incision in the skin of the neck was made and the muscle retracted to visualize the NG. Glass micropipette (WPI Single-Barrel Standard Borosilicate Glass with Filament OD1.5mm, ID0.84mm; 40µm tip beveled at 30º) attached to a picopump (WPI) was used to inject AAV into NG bilaterally (1µl/NG). For GI injections, a glass micropipette (WPI Single-Barrel Standard Borosilicate Glass with Filament OD1.5mm, ID0.84mm; 40µm tip beveled at 45º) attached to a picopump was used to deliver a series of injections into the wall of the proximal colon at a submucosal level (2 µl/rat). In a group of rats, ANGII (Bachem Cat.# H-1705, 65ng/kg/min) was delivered continuously via mini osmotic pump (Alzet 2004) as before [14].

***In vivo* calcium imaging in the NG of WKY and SHR**

*In vivo* calcium imaging was performed in the NG of anaesthetized rats as previously described with some modifications [40]. Male adult WKY and SHR were first injected with choleratoxin B (CTB, Alexa Fluor 594, Thermo Fisher) in the wall of the proximal colon, and with AAV-GCaMP6s bilaterally in the NG as described above. Three weeks later, rats were anaesthetized with isoflurane (induction at 4%; maintenance at 2% in 100% medical grade O_2_). Under anesthesia, rats were implanted with blood pressure catheters in the femoral artery, and with a separate femoral vein catheter (PE, Braintree Scientific R-FC M) that was used for delivery of increasing doses of L-phenylbiguanide (Sigma Aldrich Cat.# 164216, 0-35µg in 150µl of 0.9% NaCl). Rats were kept on a heating pad to maintain body temperature throughout the whole procedure. As described above, the NG and the vagus nerve were dissected, and the vagus nerve was cut immediately above the NG. The NG was gently placed on a stable imaging platform consisting of an 8 mm diameter coverslip attached to a metal arm affixed to a magnetic base. Surgical silicone adhesive (Kwik-Sil, WPI) was applied onto the vagus nerve to immobilize it on the coverslip, and the NG immersed in a drop of DMEM (Corning, 10-014-CV) media was covered with a second coverslip. Imaging was performed using a 16x water immersion, upright objective on a 2-Photon microscope (Bruker) fitted with a Galvanometer for acquisition, and a piezo objective combined with a galvo/resonant scanner. Images were obtained at a frame rate of 30 frames/second. Following baseline measurement of NG GCaMP6s activity and blood pressure (2 minutes), a slow i.v. infusion of L-phenylbiguanide (0-35µg in 150µl saline) was performed using a syringe pump (Harvard apparatus PHD 2000, Cat.# 70-2002) for 1 minute while simultaneously measuring the NG GCaMP6s-dependent green fluorescence and blood pressure responses in real time for 3 minutes. GCaMP6s fluorescent changes were outlined in regions of interests (ROIs) with each ROI defining a single cell throughout the imaging session. The pixel intensity in ROIs (average across pixels) was calculated frame by frame (ImageJ) and exported to Excel and GraphPad Prism for further analyses. The baseline signal was defined as the average GCaMP6s fluorescence over a 2 min period prior to the stimulus introduction. Cells were considered responsive if the following criteria were met: 1) the peak GCaMP6s fluorescence was two standard deviations above the baseline mean, and 2) the mean GCaMP6s fluorescence was 20% above the baseline mean for a 5 second window around the peak. NG in which neurons did not present baseline activity were excluded from the study. GCaMP6s responses in CTB-labeled NG neurons were analyzed separately to represent the activity of colon-projecting vagal afferents. Blood pressure responses were monitored in real time and time locked to GCaMP6s neuronal signal for correlation. At endpoint, NG were extracted and imaged with a Bruker 2-Photon microscope as above for confirmation of viral gene expression.

**Pharmacologic stimulation of 5HT3a receptors in WKY-WKY and SHR-WKY rats**

Male WKY-WKY and SHR-WKY rats were anaesthetized with isoflurane (induction at 4%; maintenance at 2% in 100% medical grade O_2_). Under anesthesia, rats were implanted with blood pressure catheters in the femoral artery, and a separate femoral vein catheter (PE, Braintree Scientific R-FC M) was used for delivery of L-phenylbiguanide (Sigma Aldrich Cat.# 164216, 35µg in 150µl of 0.9% NaCl). Following baseline measurements (2 minutes), a slow i.v. infusion of L-phenylbiguanide was performed using a syringe pump (Harvard apparatus PHD 2000, Cat.# 70-2002) for 1 minute while simultaneously measuring blood pressure and heart rate in real time. Peak responses were presented as change (Δ) from baseline for each rat and averaged within each experimental group.

**Optogenetic stimulation of NG in 5HT3aR^Cre^ rats**

Male and female 5HT3aR^Cre^ rats were injected with viral vectors expressing the control tdTomato or ChR2 either in the wall of the proximal colon, for retrograde expression, or directly in the NG as described above. Three weeks later, rats were anaesthetized (induction at 4%; maintenance at 2% in 100% medical grade O_2_) and a femoral blood pressure catheter (PE, Braintree Scientific R-FC M) was implanted, and two ECG leads were used for continuous monitoring of blood pressure and heart rate with Spike2 (CED) under anesthesia. NG was exposed bilaterally as before, and intermittent blue light (470nm, 5Hz) was applied directly to each intact NG separately for one minute while simultaneously measuring blood pressure and heart rate. Peak responses to bilateral NG stimulation were averaged for each rat and presented as change (Δ) from baseline. In one group of 5HT3aR^Cre^ rats, blue stimulating light was applied to the NG while simultaneously recording electrical activity of the vagus rostral to the NG, as previously described [79]. At endpoint, NG were extracted and imaged with a Bruker 2-Photon microscope as before for confirmation of viral gene expression.

**Restraint stress in 5HT3aR^Cre^ rats**

A group of adult male 5HT3aR^Cre^ rats were bilaterally injected in the NG with FLPo-dependent AAV expressing either Cas or YFP, and in the proximal colon wall with Cre-dependent AAVrg expressing FLPo as described above. Blood pressure and heart rate were measured at baseline and once weekly for 8 weeks as described above. Following this, rats were exposed to acute restraint stress as in [80]. Briefly, a baseline blood pressure recording was made in unrestrained rats for 20 minutes by telemetry. Following this, all rats were placed into a restrainer (Kent Scientific) for 5 minutes while simultaneously measuring blood pressure. Data was averaged for the duration of the baseline recording and compared to peak blood pressure responses under restraint. Data is presented as change (Δ) from baseline for each rat and averaged within experimental groups. At endpoint, NGs were extracted for RT-qPCR quantification of 5HT3a receptors in both groups (see methods below), and for imaging with a Bruker 2-Photon microscope to confirm viral gene expression of yellow fluorescent protein (YFP) in Control group.

**Administration of Trp diet in SHR/5HT3aR^Cre^ rats**

A group of adult male and female SHR/5HT3aR^Cre^ rats were bilaterally injected into NG with FLPo-dependent AAV expressing either 5HT3a receptor or control YFP, and into the proximal colon wall with Cre-dependent AAVrg expressing FLPo as described before. Blood pressure and heart rate were measured in all rats at baseline and then once weekly for eight weeks following viral injections as before. Following this, all rats were administered 5% Trp in diet (Research Diets, Cat.# A20012701, see Extended Data for dietary content) *ad libitum* for one week. Blood pressure and heart rate were measured by telemetry one week following Trp diet administration. Data was presented as change (Δ) from baseline for each rat and averaged within experimental groups. Food and water intake were measured once a week over 48hrs, prior and during the week of administration of Trp diet. At endpoint, NGs from both groups were extracted and imaged with a Bruker 2-Photon microscope as before for confirmation of viral gene expression as before.

**RNA Isolation and RT-qPCR in the proximal colon, NTS and NG**

RNA isolation from proximal colon, NTS and NG in rodents and human colon mucosa was performed as previously described [20, 81]. Real time quantitative PCR (RT-qPCR) was used to measure relative expression levels of 5HT3a receptors in rats using the following forward and reverse primers: *F-ACC GCC TGT AGC CTT GAC, R-TGC TCT TGT CCG ACC TCA* (IDT). Relative expression levels of Tph1 were measured in rats using the following forward and reverse primers: *F- AGC ATA ACC AGC GCC ATG AA, R- GGC ATC ATT GAC GAC ATC GAG* (IDT). Rat expression data were normalized for the expression of housekeeping gene 18S (*F-CAT TCG AAC GTC TGC CCT AT, R-GTT TCT CAG GCT CCC TCT CC*; IDT). Relative expression of human 5HT3a receptor (*F- AGC TGT CCT CCA TCC GGC AA, R- CCA GCA CCG CCA GCA GGT AAA T*; IDT) and Tph1 (*F- GCC AGT CAT CCA GGA ACA TT, R- GTT TGG CCA GAA GAT GCT GT*; IDT) were normalized to housekeeping gene β-actin (*F- AAC TAC CTT CAA CTC CAT CA, R- GAG CAA TGA TCT TGA TCT TCA*; IDT). RT-qPCR was performed using a CFX96 Touch Real-Time PCR Detection System (Bio-Rad, Hercules, CA) [20, 81]. Relative gene expression was assessed using the relative ΔΔC_t_ or alternatively ΔC_t_ method for comparisons of more than two groups, where C_t_ represented the threshold cycle.

**Immunohistology and imaging**

Tissues were post-fixed in 4% PFA (in PBS, pH=7.4) and cryopreserved in 30% sucrose for several days before being embedded in OCT Compound (Thermo Fisher Cat.# 23-730-571) and frozen at -80ºC. Tissues were sectioned with a cryostat at 35-50µm. To better visualize viral expression of YFP, some tissues were incubated with chicken anti-GFP primary antibody (1:300, Aves labs, Cat.# GFP-1010) [82, 83] followed by Goat anti-Chicken IgY Alexa Fluor 488 (1:500, Invitrogen, Cat.# A-11039) . For 5-HT staining in the colon, primary rabbit anti-5-HT (1:500, Immunostar, Cat.# 20080) and anti-rabbit secondary antibody (1:1000, Invitrogen, A-21245) were used. Sections were first washed in Tris-buffered saline (TBS) with 0.05% Tween-20, followed by incubation with 10% normal serum. Tissues were incubated with appropriate primary antibodies overnight at 4°C, then rinsed in TBS/0.05% Tween-20 solution, followed by incubation with secondary antibodies for 40-60 minutes at room temperature. Slides were mounted (Abcam, Cat.# ab104139) and cover slipped. Fluorescence imaging was performed with the Leica Microsystems TCS SP5 multi-photon laser scanning confocal microscope (10-40x objective lens, 1-5µm z step size). Pseudo-coloring from green to yellow was performed in ImageJ in tissues expressing viral YFP for clarity.

**Statistical analyses**

Sample sizes were determined according to standard power analysis (80% power and α = 0.05) based on Cohen’s d standardized effect sizes as in previous reports [10, 14]. Animals were randomly assigned to treatment groups. Whenever feasible, data collection and/or analyses were performed by a blinded experimenter. After testing for normality (Shapiro-Wilk test), intergroup differences were analyzed by unpaired or paired, two-tailed Student’s t-test for single comparison, or by one-way or two-way analysis of variance (ANOVA) with an appropriate multiple-comparison test, as indicated in the figure legends. If nonparametric testing was indicated, intergroup differences were analyzed by Mann-Whitney U-test or Kruskal-Wallis test with Dunn’s correction, as appropriate. The F test for equality of variances was performed and, if indicated, appropriate corrections were made (Welch’s correction for the t-test) using Prism 9 (GraphPad). Longitudinal telemetry data (repeated blood-pressure measurements in the same animals over time; e.g., Fig. 1C, 3I, 5F and 6D) were analyzed using a mixed-effects model (restricted maximum likelihood) equivalent to a two-way repeated-measures ANOVA, with treatment (group) and time as factors and a random intercept for individual animal to account for within-subject correlation across weeks; a mixed-effects model was used rather than classical repeated-measures ANOVA so that animals with an occasional missing weekly time point are retained. Significant main effects or interactions were followed by Sidak-corrected multiple comparisons at each time point. Sample sizes were determined a priori by power analysis (80% power, α=0.05) based on the expected blood-pressure effect size (Cohen’s d) from our prior work. The experimental unit was the individual animal throughout; where multiple measurements were obtained per animal (e.g., left and right nodose ganglia), values were averaged so that the animal, not the ganglion or neuron, served as the unit of analysis. A total of 120 animals were used in this study; per-experiment group sizes are reported in each figure legend. The statistical reporting in this work follows the ARRIVE 2.0 guidelines [15]. Data are presented as mean ± SEM.

**Data availability**

Any additional data requests are available from the corresponding authors upon request.

**Table S1. List of viral vectors**

| **Viral vector** | **Abbreviation** | **Vendor** | **Catalogue Number** |
| --- | --- | --- | --- |
| AAV9-EF1a-fDIO-taCasp3-TEVp-WPRE | AAV-fDIO-Cas | Vector Biolabs | Custom made (see vector map below) |
| AAV9-Ef1a-fDIO EYFP | AAV-fDIO-YFP | Addgene | 55641-AAV9 |
| AAVrg-EF1a-DIO-FLPo-WPRE-hGHpA | AAVrg-DIO-FLPo | Addgene | 87306-AAVrg |
| AAV9-Syn-GCaMP6s-WPRE-SV40 | AAV-GCAMP6s | Addgene | 100843-AAV9 |
| AAV9-EF1a-DIO-tdTomato | AAV-DIO-tdTomato | Addgene | 28306-AAV9 |
| AAV9-Ef1a-fDIO-5htr3a-2A-eGFP | AAV-fDIO-5ht3aR | Vector Biolabs | Custom made  (see vector map below) |
| AAVrg-EF1a-DIO-tdTomato | AAVrg-DIO-tdTomato | Addgene | 28306-AAVrg |
| AAVrg-EF1a-DIO-hChR2(H134R)-eYFP-WPRE-HGHpA | AAVrg-DIO-ChR2 | Addgene | 20298-AAVrg |
| AAV5-flex-taCasp3-TEVp | AAV-DIO-Cas | Addgene | 45580-AAV5 |
| AAV9-EF1a-DIO-hChR2(H134R)-eYFP-WPRE-HGHpA | AAV-DIO-ChR2 | Addgene | 20298-AAV9 |

**Vector maps**

**Table S2. List of antibodies**

| **Antibody** | **Host** | **Vendor** | **Catalogue Number** | **Type** |
| --- | --- | --- | --- | --- |
| GFP | Chicken | Aves Labs | GFP-1020 | Primary |
| Anti-Chicken IgY (H+L) Alexa Fluor^TM^ 488 | Goat | Invitrogen | A-11039 | Secondary |

**Table S3. Trp diet full content**

| **Product #** | **A20012701** | |
| --- | --- | --- |
|  | **5% Trp** | |
|  | gm% | kcal% |
| Protein | 22% | 23% |
| Carbohydrate | 64% | 66% |
| Fat | 5% | 12% |
| Total |  | 100% |
| kcal/gm |  |  |
|  |  |  |
| Ingredient (gm) | gm | kcal |
| L-Arginine | 10 | 40 |
| L-Histidine-HCl-H2O | 6 | 24 |
| L-Isoleucine | 8 | 32 |
| L-Leucine | 12 | 48 |
| L-Lysine-HCl | 14 | 56 |
| L-Methionine | 6 | 24 |
| L-Phenylalanine | 8 | 32 |
| L-Threonine | 8 | 32 |
| L-Tryptophan | 50 | 200 |
| L-Valine | 8 | 32 |
| L-Alanine | 10 | 40 |
| L-Asparagine-H2O | 5 | 20 |
| L-Aspartate | 10 | 40 |
| L-Cystine | 4 | 16 |
| L-Glutamic Acid | 30 | 120 |
| L-Glutamine | 5 | 20 |
| Glycine | 10 | 40 |
| L-Proline | 5 | 20 |
| L-Serine | 5 | 20 |
| L-Tyrosine | 4 | 16 |
|  |  |  |
| Corn Starch | 502.5 | 2010 |
| Maltodextrin 10 | 125 | 500 |
| Cellulose | 50 | 0 |
|  |  |  |
| Corn Oil | 50 | 450 |
|  |  |  |
| Mineral Mix S10001 | 35 | 0 |
| Sodium Bicarbonate | 7.5 | 0 |
|  |  |  |
| Vitamin Mix V10001 | 10 | 40 |
| Choline Bitrartrate | 2 | 0 |
|  |  |  |
| Yellow Dye, FD&C #5 | 0.05 | 0 |
| Red Dye, FD&C #40 | 0 | 0 |
| Blue Dye, FD&C #1 | 0 | 0 |
|  |  |  |
| **Total** | **1000.05** | **3872** |
|  |  |  |
| % Tryptophan | 5.00 |  |

**Figure S1: Hypertensive microbiota increased blood pressure and reduced 5HT3a receptor expression along the vagal gut-brain axis with no change in fecal serotonin**

**A,** five days of oral gavage of a cocktail of antibiotics (ABX) in Wistar-Kyoto (WKY) rats significantly reduced fecal bacterial DNA mass (by~75%), confirming endogenous bacterial depletion prior to fecal microbiota transplant (FMT). Values are means ± SEM; N=5/group; Student’s Ttest, ****P<0.0001.

**B**, spontaneously hypertensive rats (SHR, light red) microbiota donors had significantly higher systolic blood pressures (SBP, left panel) and lower colonic serotonin (right panel) as measured by ELISA, than the Wistar-Kyoto (WKY, light blue) microbiota donors. Values are means ± SEM; N=5/group; Student’s Ttest, *P<0.05, ****P<0.0001.

**C**, reduced relative expression levels of 5-HT3a receptors (5HT3aR) in the nodose ganglia (NG, left panel), nucleus of the solitary tract (NTS, middle panel) and colon (right panel) in SHR donors (light red) and the WKY recipients of FMT from the SHR (SHR-WKY, dark red), compared to the Wistar-Kyoto (WKY, light blue) microbiota donors and the recipients of FMT from the WKY (WKY-WKY, dark blue). Values are means ± SEM; N=5/group; one-way ANOVA followed by Sidak's multiple-comparisons test (or Kruskal-Wallis with Dunn's correction where data did not meet normality), *P<0.05, **P<0.01, ***P<0.001, ****P<0.0001.

**D**, top left, cartoon schematics of untargeted metabolomics performed in fecal bacterial samples of WKY-WKY (blue) and SHR-WKY (red) groups using LC-MS/MS at endpoint (Week 8 post-FMT). The panel below (Volcano plot) and the middle panel (heat map) reflect significant divergence of overall metabolites between the WKY-WKY (blue) and SHR-WKY (red) (N=3-4/group). Far right panel illustrates no significant difference in serotonin (5-HT) measured by ELISA in fecal bacterial samples of WKY-WKY (blue) and SHR-WKY (red) group (N=5/group). Values are means ± SEM; Student’s Ttest.

**E**, Radiotelemetric measurements of continuous blood pressures (BP, mmHg) and heart rate (HR, bpm-beats per minute) performed once a week over 24hrs in conscious unrestrained WKY-WKY (blue) and SHR-WKY (red) rats at baseline (i.e., before fecal microbiota transplant (FMT), noted as wk0) and then at weeks 4-8. Line across all time points reflect significant interaction between treatment and time. Values are means ± SEM; mixed-effects model (REML) with treatment and time as factors and animal as a random effect, followed by Sidak's multiple-comparisons test, *P<0.05, **P<0.01.

**Figure S2: Normotensive microbiota transplant decreased blood pressure and increased colonic serotonin in the SHR.**

**A,** five days of oral gavage of a cocktail of antibiotics (ABX) in juvenile (8wo) male spontaneously hypertensive rats (SHR) followed by whole fecal microbiota transplant (FMT) from either the normotensive Wistar-Kyoto (WKY) or SHR donors (adult, male) to create the two experimental FMT groups.

**B**, At 8 weeks post-FMT, the group receiving FMT from WKY microbiota donors had significantly lower systolic blood pressures (SBP, blue bars, WKY-SHR) than the group receiving FMT from SHR microbiota donors (red bars, SHR-SHR).

**C**, 16S sequencing demonstrates significant separation of gut bacterial profiles between the two FMT groups confirming FMT.

**D**, higher colonic serotonin levels in the WKY-SHR group were observed at endpoint (week 8 post-FMT) as measured by ELISA.

**E**, higher relative expression levels of tryptophan hydroxylase 1 (Tph1) and 5HT3a receptors (5HT3aRs) were observed in the colons of WKY-SHR compared to the SHR-SHR group at endpoint (week 8 post-FMT), as measured by qPCR.

**F**, higher relative expression levels of 5HT3aRs were observed in the nodose ganglia (NG) and the nucleus of the solitary tract (NTS) of WKY-SHR compared to the SHR-SHR group at endpoint (week 8 post-FMT), as measured by qPCR. Values are means ± SEM; N=4/group; Student’s Ttest, *P<0.05.

**Figure S3: Hypertensive microbiota epigenetically regulates colonic Tph1.**

**A-B,** Chromatin-immunoprecipitation quantitative polymerase chain reaction (ChIP-qPCR) assays were performed on colons using an antibody to detect acetylation at histone H3, lysine 9 (H3K9ac) or a control IgG antibody. Acetylation levels at the *Tph1* and *Tph2* promoter regions were quantified by ChIP-qPCR using gene-specific primers. **A**, acetylation levels at the *Tph1* and *Tph2* promoter regions in colons from naïve WKY and SHR donors. **B**, acetylation levels at the *Tph1* and *Tph2* promoter regions in colons from fecal matter transplant (FMT) recipients.

**C**, quantitative real time PCR (RT-qPCR) analysis of relative expression levels of tryptophan hydroxylase 1 (Tph1) normalized to 18S housekeeping gene in the proximal colons of the donor and FMT recipient groups. Values are means ± SEM; N=5/group; one-way ANOVA followed by Sidak's multiple-comparisons test (or Kruskal-Wallis with Dunn's correction where data did not meet normality), *P<0.05, **P<0.01, ***P<0.001.

**Figure S4. Chronic ablation of vagal afferents expressing 5HT3a receptors increases mean and diastolic blood pressure**

**A,** proximal colons from Control-Cre virus-injected 5HT3aRCre rats were sectioned and imaged using fluorescence microscopy. tdTomato fluorescent labeling of vagal afferent projections was observed in proximal colons of Control-injected rats. Scale bar =100 µm.

**B**, nodose ganglia image from a naïve (non-Cre) adult male Long-Evans rat injected with the Cre-dependent virus expressing GFP shows no fluorescent expression in the nodose, thus confirming the specificity of viral Cre-dependent approach. Scale bar =50 µm.

**C,** a significant increase in mean blood pressure (MBP, by~20mmHg, left panel) and diastolic blood pressure (DBP, by ~20mmHg, middle panel) was observed in 5HT3aR^Cre^ rats at wk8 following bilateral nodose ganglia microinjection of viral vector expressing Caspase 3 (Caspase-ablated, magenta) compared to the Control group (gray) (N=5/group). Line across all time points reflects significant interaction between treatment and time for MBP and DBP. No significant difference was observed in HR (right panel) between the groups at any time point. Absolute values are normalized to values observed at baseline for each rat and presented as change (Δ) from baseline (wk0). Values are means ± SEM; mixed-effects model (REML) with treatment and time as factors and animal as a random effect, followed by Sidak's multiple-comparisons test, *P<0.05.

**Figure S5: Reduced serotonin signaling by intestinal vagal afferents in hypertensive rodents with gut dysbiosis**

**A,** heat maps depict z-score-normalized fluorescence traces from all GCaMP6s^+^ vagal neurons in the nodose ganglia of normotensive Wistar-Kyoto (WKY) rats (left panel) and spontaneously hypertensive rats (SHR, right panel), responding to i.v. infusion of serotonin receptor agonist L-phenylbiguanide (3.5µg). Each row represents the activity of a single cell over 5 mins. Stimulus was given at 120 seconds (white box). In N=5 per group, 34% of the total of 138 GCaMP6s-expressing neurons responded to the serotonin receptor agonist in the WKY rats, with only 7% of 57 GCaMP6s-expressing neurons were responsive in the SHR, as depicted by pie charts. The responses of each neuron are represented by heat maps.

**B**, heat maps depict z-score-normalized fluorescence traces only from colon-projecting GCaMP6s^+^ vagal neurons in the nodose ganglia of normotensive Wistar-Kyoto (WKY) rats responding to i.v. infusion of serotonin receptor agonist (3.5µg). Each row represents the activity of a single cell over 5 mins. Stimulus was given at 120 seconds (white box). In N=5 per group, 29% of the total of the 41 colon-projecting GCaMP6s^+^ vagal neurons responded to the serotonin receptor agonist in the WKY rats, while none of the 15 colon-projecting GCaMP6s^+^ neurons were responsive in the SHR, as depicted by pie charts. The responses of each neuron are represented by heat maps.

**C,** heat maps depict z-score-normalized fluorescence traces from all GCaMP6^+^ vagal neurons in the nodose ganglia of normotensive Wistar-Kyoto (WKY) rats (left panel) and spontaneously hypertensive rats (SHR, right panel), responding to i.v. infusion of serotonin receptor agonist (0.35µg). Each row represents the activity of a single cell over 5 mins. Stimulus was given at 120 seconds (white box). In N=5 per group. 16% of the total of 238 GCaMP6s^+^ vagal neurons responded to the serotonin receptor agonist in the WKY rats, while only 2% of 162 GCaMP6s^+^ vagal neurons were responsive in the SHR, as depicted by pie charts. The responses of each neuron are represented by heat maps.

**D**, heat maps depict z-score-normalized fluorescence traces only from colon-projecting GCaMP6s^+^ vagal neurons in the nodose ganglia of normotensive Wistar-Kyoto (WKY) rats (left panel) responding to i.v. infusion of serotonin receptor agonist (0.35µg). Each row represents the activity of a single cell over 5 mins. Stimulus was given at 120 seconds (white box). In N=5 per group, 8% of the total of the 63 colon-projecting GCaMP6s^+^ vagal neurons responded to the serotonin receptor agonist in the WKY rats, while none of the 67 colon-projecting GCaMP6s^+^ vagal neurons were responsive in the SHR, as depicted by pie charts. The responses of each neuron are represented by heat maps.

**Figure S6: Viral ablation of colon-projecting serotonin-sensing vagal neurons elevates blood pressure**

**A,** endpoint confirmation of viral expression of fluorescence reporters in the nucleus of the solitary tract (NTS, top panel, scale bar = 300 µm), proximal colon (bottom panel, scale bar = 300 µm), and in the nodose ganglia of Control (left, with the inset image showing no green fluorescence in Control NG) and ChR2 (right, scale bar=100µm) in 5HT3aR^Cre^ rats injected with retrograde AAVrg in the proximal colon. AP-area postrema; CC-central canal.

**B,** a representative image of viral expression of fluorescent reporter in colon-projecting vagal afferents in the nodose ganglia. The images are representative of viral transfection in the Control YFP group only, as the Caspase AAV construct does not express a fluorescent reporter (see details on viral constructs in Extended Data). Scale bar = 75µm.

**C,** radiotelemetric measurements of continuous mean BP (MBP, mmHg), diastolic BP (DBP, mmHg) and heart rate (HR, beats per minute, bpm) were performed once a week over 24hrs in conscious unrestrained 5HT3aR^Cre^ rats at baseline (i.e., before nodose ganglia viral injections, noted as wk0) and then at weeks 4-8. A significant increase in MBP (by~8mmHg, left panel) was observed in the Caspase-ablated group (magenta) compared to Controls (gray) and to its own baseline (wk0) at wk8 post injection. In addition, a significant increase in DBP (by ~8mmHg, middle panel) was observed in the Caspase-ablated group (magenta) at wk8 post-injection compared to its baseline (wk0). Lastly, a significant decrease in HR (by ~20-25 bpm) was observed at all time points in the Control (gray) but not the Caspase-ablated group (magenta). Absolute values are normalized to values observed at baseline for each rat and presented as change (Δ) from baseline. N=5/group. Values are means ± SEM; mixed-effects model (REML) with treatment and time as factors and animal as a random effect, followed by Sidak's multiple-comparisons test, *P<0.05.

**D**, endpoint 16S bacterial sequencing analyses show an overlap in composition and abundances of the gut bacteria between the two groups, illustrated by the principal coordinate analysis (PCoA) showing beta diversity with Bray-Curtis Dissimilarity and Alpha diversity plots with the common indices (far right).

**Figure S7. Viral expression of serotonin receptors in intestinal vagal afferents decreases blood pressure in hypertensive SHR/5HT3aR^Cre^ rats with gut dysbiosis**

**A,** cartoon schematic depicting generation of the SHR/5HT3aR^Cre^ model. Female SHR were bred to male LE-5ht3a*^em1(T2A-Cre)Sage^* (5HT3aR^Cre^) rats. Following weaning, the offspring were transplanted with SHR gut microbiota in bedding, twice weekly until adulthood.

**B**, adult SHR/5HT3aR^Cre^ rats presented with significantly higher systolic blood pressures (SBP, mmHg) compared to the 5HT3aR^Cre^ controls (left panel) and trending towards the SHR. On the right, principal coordinate analysis (PCoA) showing beta diversity following 16S sequencing of fecal bacteria revealed overlapping microbiota profiles between the SHR donors (light red) and the SHR/5HT3aR^Cre^ offspring recipients of the SHR microbiota (dark gray). In addition, a significant separation in the gut microbiota composition and abundances was demonstrated between the SHR/5HT3aR^Cre^ model (dark gray) and the 5HT3aR^Cre^ controls (light gray).

**C**, top left shows no difference in the food intake before and during administration with 5% Trp in diet between the groups. Radiotelemetric measurements of continuous mean BP (MBP, mmHg), diastolic BP (DBP, mmHg) and heart rate (HR, beats per minute, bpm) were performed once a week over 24hrs in conscious unrestrained SHR/5HT3aR^Cre^ rats at baseline (i.e., before nodose ganglia viral injections noted as wk0) and then at weeks 4-9. Absolute values are normalized to values observed at baseline for each rat and presented as change (Δ) from baseline. N=4-6/group. Values are means ± SEM; mixed-effects model (REML) with treatment and time as factors and animal as a random effect, followed by Sidak's multiple-comparisons test, *P<0.05, **P<0.01.
